# Interleukin-11 is important for vascular smooth muscle phenotypic switching and aortic inflammation, fibrosis and remodeling in mouse models

**DOI:** 10.1038/s41598-020-74944-7

**Published:** 2020-10-20

**Authors:** Wei-Wen Lim, Ben Corden, Benjamin Ng, Konstantinos Vanezis, Giuseppe D’Agostino, Anissa A. Widjaja, Wei-Hua Song, Chen Xie, Liping Su, Xiu-Yi Kwek, Nicole G. Z. Tee, Jinrui Dong, Nicole S. J. Ko, Mao Wang, Chee Jian Pua, Muhammad H. Jamal, Beeyong Soh, Sivakumar Viswanathan, Sebastian Schafer, Stuart A. Cook

**Affiliations:** 1 National Heart Research Institute Singapore, National Heart Centre Singapore, Singapore, 169609 Singapore; 2 Cardiovascular and Metabolic Disorders Program, Duke-National University of Singapore Medical School, 8 College Road, Singapore, 169857 Singapore; 3 MRC-London Institute of Medical Sciences, Hammersmith Hospital Campus, London, W12 0NN UK; 4 National Heart and Lung Institute, Imperial College London, London, SW3 6LY UK

**Keywords:** Vascular diseases, Experimental models of disease, Interleukins, Transforming growth factor beta, Cell biology

## Abstract

Transforming growth factor beta-1 (TGFβ1) is a major driver of vascular smooth muscle cell (VSMC) phenotypic switching, an important pathobiology in arterial disease. We performed RNA-sequencing of TGFβ1-stimulated human aortic or arterial VSMCs which revealed large and consistent upregulation of *Interleukin 11* (*IL11)*. IL11 has an unknown function in VSMCs, which highly express the IL11 receptor alpha, suggestive of an autocrine loop. In vitro, IL11 activated ERK signaling, but inhibited STAT3 activity, and caused VSMC phenotypic switching to a similar extent as TGFβ1 or angiotensin II (ANGII) stimulation. Genetic or therapeutic inhibition of IL11 signaling reduced TGFβ1- or ANGII-induced VSMC phenotypic switching, placing IL11 activity downstream of these factors. Aortas of mice with *Myh11*-driven IL11 expression were remodeled and had reduced contractile but increased matrix and inflammatory genes expression. In two models of arterial pressure loading, IL11 was upregulated in the aorta and neutralizing IL11 antibodies reduced remodeling along with matrix and pro-inflammatory gene expression. These data show that IL11 plays an important role in VSMC phenotype switching, vascular inflammation and aortic pathobiology.

## Introduction

Vascular smooth muscle cells (VSMCs) are specialized cells found within the medial layer of the vasculature. In health, VSMCs proliferate at a low rate, maintain their cellular identity and express high levels of contractile proteins, consistent with their role in preserving physiological vascular tone. In disease, VSMCs lose their contractile properties and differentiate into a synthetic phenotype mainly characterized by secretion of extracellular matrix, increased proliferation and migration^1^. This cellular transition is termed *phenotypic switching* and plays a key role in the pathophysiology of adverse aortic remodeling in hypertension, aneurysm development and atherosclerosis^2–8^.

Two key cytokines associated with VSMC phenotypic switching and adverse aortic remodeling are transforming growth factor-beta (TGFβ) and angiotensin-II (ANGII)^9–12^. It is notable that both TGFβ gain- and loss-of-function can be associated with aortic remodeling due to the pleiotropic effects of TGFβ across cell types, thus highlighting an incomplete understanding of VSMC pathobiology^10,13^. Fibroblast-to-myofibroblast transition and VSMC phenotypic switching share many molecular features such as collagen secretion and cell migration and can be triggered by identical stimuli. We recently discovered that IL11 is important for fibroblast activation downstream of both TGFβ1 and ANGII, which begs the question as to whether IL11 signaling is also important for VSMC biology^14–16^.

Little is known about the function of IL11 in VSMCs. A study from 1999 showed that both interleukin-1 alpha (IL1α) and TGFβ1 dose-dependently and synergistically induce IL11 secretion from human aortic VSMCs^17^. In 2002, IL11 was reported to inhibit FGF-induced VSMC proliferation^18^. More recent microarray studies showed that IL11 is one of the most highly upregulated genes in human coronary artery VSMCs stimulated with TGFβ1 and that both IL1α and TGFβ1 induce IL11 secretion in these cells^19^. Outside of the vasculature, IL11 has been linked with smooth muscle cell proliferation in the lung^20^.

Given the similarities of the cellular and molecular properties of both myofibroblasts and synthetic VSMCs, and the common stimuli driving these phenotypes, we hypothesized that IL11 may be important for VSMC phenotypic switching. We examined this premise in primary human VSMCs stimulated with IL11, ANGII or TGFβ1 along with IL11 loss-of-function, using a range of in vitro assays. We also assessed the potential contribution of IL11 to aortic remodeling in vivo using transgenic mice with smooth muscle cell (SMC)-restricted Il11 expression and in two models of pressure overload where we examined the effects of neutralizing IL11 antibodies on aortopathy.

## Results

### TGFβ1 stimulation of VSMCs causes phenotypic switching

Primary human VSMCs were derived from aortic and left internal mammary artery (LIMA) biopsies. Detailed patient demographics are illustrated in Supplementary Table S1 online. To purify VSMCs, we negatively selected cells against cluster of differentiation 90 (CD90)^+ve^ fibroblasts and CD144^+ve^ endothelial cells. Cultured VSMCs had high expression levels of contractile proteins (F-actin, myosin light chain, smooth muscle myosin heavy chain, and transgelin) and no detectable endothelial or fibroblast markers (Supplementary Fig. S1 online).

To characterize the biological effects of TGFβ1 on VSMCs, we performed RNA-seq of stimulated and unstimulated cells from 27 donors in total (10 aortic, 22 LIMA, of which 5 aortic and 5 LIMA tissue were paired samples from the same donors). A total of 3567 differentially expressed genes were identified in arterial VSMCs and 2108 differentially expressed genes in aortic VSMCs in paired samples after TGFβ1 treatment (Supplementary Fig. S2 online). Of these genes, 1,560 were common to both types of VSMCs and significantly enriched with protein synthesis and secretion-related gene ontology (GO) terms such as ‘vesicle-mediated transport’, ‘secretion by cell’, ‘collagen metabolic process’ or ‘extracellular matrix structural constituent’. Furthermore, gene set enrichment analysis (GSEA) (Supplementary Fig. S3 online) showed enrichment of GO and Hallmark gene categories indicative for the epithelial-to-mesenchymal transition, motility and the secretory machinery. As expected, global transcriptome profiling suggests gene expression changes related to TGFβ1-induced phenotypic switching VSMCs.

### TGFβ1 induces IL11 secretion from VSMCs

**Figure 1:**
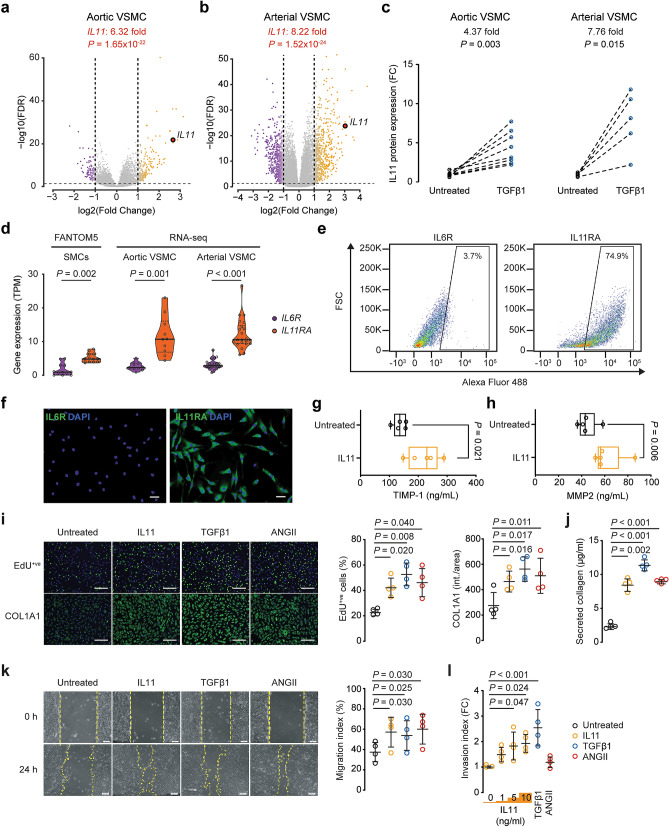
IL11 is secreted from VSMCs stimulated with TGFβ1 and causes VSMC phenotypic switching. Volcano plots of RNA-seq analysis showing the transcriptomic response of (**a**) aortic and (**b**) arterial VSMCs to TGFβ1 stimulation (Aortic, *n* = 10; left internal mammary artery (LIMA), n = 22). FDR: false discovery rate. (**c**) ELISA of IL11 in the supernatant of VSMC cultures following TGFβ1 stimulation (*n* = 5–8 biological replicates). (**d**) From left to right, *IL6R* and *IL11RA* gene expression in SMCs (*n* = 13, FANTOM5 database), RNA-seq data from unstimulated aortic (*n* = 10) and arterial (*n* = 22) VSMCs. 1 outlier was removed from the FANTOM5 dataset by the 2% ROUT method. Data presented as violin plots with quartiles indicated by dotted lines and median indicated by full lines. TPM: transcripts per million. (**e**) Flow cytometry forward scatter (FSC) plots of arterial VSMCs at baseline demonstrated IL11RA to be highly expressed in VSMCs whereas IL6R expression is scant. (**f**) Representative immunofluorescence staining of VSMCs for IL11RA and IL6R showing high IL11RA and undetectable IL6R expression. Stimulation of human VSMCs with IL11 (*n* = 5 biological replicates) increases both (**g**) TIMP1 and (**h**) MMP2 secretion. (**i**) Proliferation (EdU^+ve^ cells) and COL1A1 expression by human VSMCs following IL11, TGFβ1 or ANGII stimulation (representative immunostaining images and Operetta assay results from 4 independent experiments). (**j**) Collagen secretion as measured by Sirius red collagen detection assay in culture medium following cytokine stimulation (*n* = 4). (**k**) VSMC migration measured by scratch wound assay following cytokine stimulation (*n* = 4). (**l**) Matrigel invasion indices of VSMCs induced by IL11 (1, 5, and 10 ng/ml), TGFβ1 or ANGII (*n* = 4). FC: fold change. Scale bars in representative images for **f** represent 100 µm and **i** and **k** represent 200 µm. Statistical analyses by two-tailed paired *t*-tests or one-way ANOVA with Sidak post hoc tests; data either presented as median ± IQR with whiskers demarcating minimum and maximum values or mean ± SD. Cytokines were applied for 24 h at doses of 5 ng/ml for IL11 and TGFβ1 and 100 nM for ANGII, unless otherwise stated.

VSMC phenotypic switching was also characterized by large upregulation of the *IL11* gene in primary human cells (Aortic: 6.32-fold, *P* = 1.65 × 10^–22^; Arterial: 8.22-fold, *P* = 1.52 × 10^–24^; Fig. 1a,b). In response to TGFβ1 treatment (5 ng/ml, 24 h), *IL11* was the 5^th^ and 9^th^ most upregulated gene in aortic and arterial VSMCs respectively (Supplementary Table S2 online). ELISA assays confirmed that this was forwarded to the protein level, resulting in significantly increased IL11 protein secretion from TGFβ1 stimulated VSMCs (Aortic: 4.37-fold, *P* = 0.003; Arterial: 7.76-fold, *P* = 0.015; Fig. 1c). Another driver of phenotypic-switching, ANGII^12,21^, also significantly increased IL11 secretion from VSMCs by 2.03-fold (*P* = 0.038; Supplementary Fig. S4 online).

### The IL11 receptor alpha (IL11RA) is highly expressed in VSMCs

We explored the expression of IL11RA in VSMCs and compared it to *interleukin 6 receptor* (*IL6R)*, which also signals through glycoprotein 130 (gp130). Cap analysis gene expression (CAGE) data of SMCs in the FANTOM5 database^22^, as well as our own RNA-seq data from aortic and arterial VSMCs, revealed that SMCs express significantly more *IL11RA* transcripts compared to *IL6R* (FANTOM5: 2.19-fold difference in transcripts per million (TPM), *P* = 0.002; Aortic: 4.54-fold difference in TPM, *P* = 0.001; Arterial: 3.94-fold difference in TPM, *P* < 0.001; Fig. 1d). At the protein level, flow cytometry and immunofluorescence showed high expression of IL11RA in VSMCs (Fig. 1e,f, Supplementary Fig. S5 online). In contrast, IL6R was hardly detected in primary human VSMCs consistent with this cell type being unresponsive to direct IL6 stimulation^23^.

### IL11 stimulates VSMC phenotypic switching

We have previously shown that IL11 elicits its effects in fibroblasts primarily through regulation of gene translation but not transcription^14^. Like fibroblasts, we observed very little effects of IL11 stimulation (5 ng/ml, 24 h) on the transcriptome in VSMCs with no significantly differentially expressed genes (> twofold and FDR < 0.05) compared to unstimulated VSMCs (See Supplementary Fig. S6 online). Similarly, RT-qPCR for *ACTA2*, *CNN1*, *MYOCD*, *SM22α*, and *MYH11* contractile and *COL1A1*, and *COL3A1* collagen gene expression in human VSMCs stimulated with IL11 (5 ng/ml, 24 h) confirmed no differences in gene expression (See Supplementary Fig. S6 online).

IL11 induces the production and secretion of tissue inhibitor matrix metalloproteinase 1 (TIMP1) and matrix metalloproteinase 2 (MMP2) from fibroblasts^14^ and hepatic stellate cells^15^, contributing to extracellular matrix remodeling and fibrosis. In VSMCs, MMP2 is involved in phenotypic switching^24^ and migratory properties^25^, whereas TIMP1 is upregulated by fibrogenic stimuli^26^ and induced VSMC proliferation^27^. Similarly, stimulating VSMCs with IL11 (5 ng/ml, 24 h) also significantly increased TIMP1 and MMP2 secretion (*P*_*TIMP1*_ = 0.021, *P*_*MMP2*_ = 0.006; Fig. 1g,h). Coupled with a reduction in expression of contractile markers SM22α and MYOCD (See Supplementary Fig. S7 online), this suggests that IL11 promotes phenotypic switching in VSMCs towards a less contractile and more synthetic phenotype.

We next compared the effects of IL11 on VSMCs to TGFβ1 or ANGII, which are widely recognized as major determinants of VSMC cellular transitions. VSMCs incubated with either stimulus showed significant increases in EdU^+ve^ proliferation (*P*_IL11_ = 0.020, *P*_TGFβ1_ = 0.008, *P*_ANGII_ = 0.040; Fig. 1i), COL1A1 expression (*P*_IL11_ = 0.016, *P*_TGFβ1_ = 0.017, *P*_ANGII_ = 0.011) and total collagen secretion (*P*_IL11_ = 0.002, *P*_TGFβ1_ < 0.001, *P*_ANGII_ < 0.001; Fig. 1j) with the magnitude of the IL11 effect similar to ANGII but lesser than TGFβ1.

In the scratch wound migration assay, IL11 induced VSMC migration to a similar extent as TGFβ1 or ANGII stimulation (*P*_IL11_ = 0.030, *P*_TGFβ1_ = 0.025, *P*_ANGII_ = 0.030; Fig. 1k). In Matrigel invasion assays, stimulation of VSMCs with increasing doses of IL11 (24 h, 1–10 ng/ml) resulted in significantly more invasive activity as compared with unstimulated controls. In our hands, ANGII stimulation did not significantly increase VSMC invasiveness, perhaps reflecting its bimodal effects on this phenotype^28^ (Fig. 1l). Taken together these data imply a role for IL11 in VSMC phenotypic switching.

### IL11 is required for TGFβ1- or ANGII-stimulated VSMC phenotypic switching

IL11 is upregulated by TGFβ1 or ANGII and sufficient on its own to induce the VSMC phenotype transition. To test whether IL11 signaling is required downstream of TGFβ1 or ANGII-driven VSMC phenotypic switching, we cultured aortic VSMCs from *Il11ra1*^*−/−*^ or *Il11ra1*^+*/*+^ mice. Similar to our observations in human VSMCs, *Il11ra1*^+*/*+^ VSMCs displayed increased migration and collagen secretion with IL11, TGFβ1 or ANGII stimulation. In contrast to this, in VSMCs from *Il11ra1*^*-/-*^ mice, both the migratory response and secreted collagen concentration to all three stimuli was significantly reduced (Migration: *P*_IL11_ = 0.006, *P*_TGFβ1_ < 0.001, *P*_*ANGII*_ = 0.008; Secreted collagen: *P*_IL11_ = 0.003, *P*_TGFβ1_ < 0.001, *P*_*ANGII*_ = 0.004; Supplementary Fig. S8 online).

**Figure 2:**
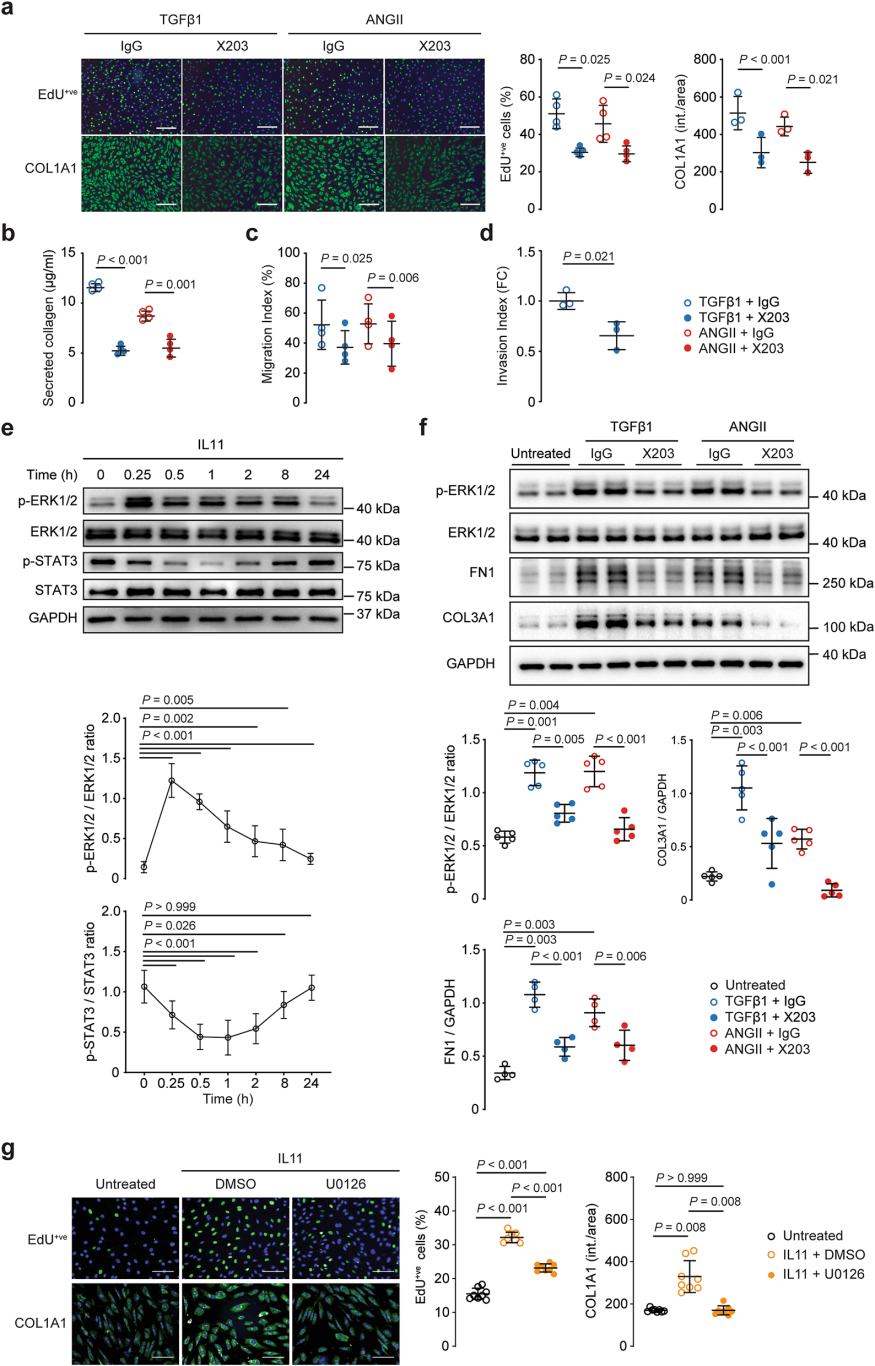
Inhibition of VSMC phenotypic switching with neutralizing IL11 antibodies or ERK inhibition. (**a**) Proliferation (EdU^+ve^ staining) and COL1A1 expression after TGFβ1 or ANGII stimulation in the presence of isotype control (IgG) or IL11 (X203) antibodies. Representative immunostaining images and Operetta assay results from 4 independent experiments. (**b**) Total collagen secretion (Sirius Red) following TGFβ1 or ANGII stimulation with either IgG or X203 antibody (*n* = 4). (**c**) Cytokine-induced VSMC migration (scratch wound assay) in the presence of IgG or X203 (*n* = 4). (**d**) Matrigel invasion indices of TGFβ1-stimulated VSMC in the presence of X203 or IgG antibody (*n* = 3). (**e**) Representative immunoblots of IL11-induced ERK1/2 and STAT3 phosphorylation in human VSMCs. Samples were derived from the same experiment and blots were processed in parallel. Extended blots are presented in Supplementary Fig. S12 online. Collated densitometry for phosphorylated ERK1/2 and STAT3 normalized to total ERK1/2 and STAT3 respectively (*n* = 9). *P*-values stated in comparison to 0 min time point. (**f**) Representative immunoblots of ERK1/2 activation and extracellular matrix proteins fibronectin (FN1) and collagen 3 (COL3A1) in human VSMCs stimulated with TGFβ1, or ANGII in the presence of anti-IL11 (X203) or IgG control antibodies. Samples were derived from the same experiment and blots were processed in parallel. Extended blots are presented in Supplementary Fig. S12 online. Collated densitometry for phosphorylated ERK1/2 normalized to total ERK1/2, FN1 and COL3A1 normalized to GAPDH (*n* = 5). (**g**) Proliferation (EdU^+ve^ cells) and COL1A1 expression after IL11 stimulation and either MEK/ERK inhibitor U0126 (10 µM) or dimethyl sulfoxide (DMSO) vehicle. Representative immunostaining images and Operetta assay results from 4 independent experiments. Scale bars in representative images for **a** represent 200 µm and **g** represent 100 µm. Statistical analyses by one-way ANOVA with Sidak post hoc tests or two-tailed paired *t*-test; data presented as mean ± SD. Cytokines were applied for 24 h at doses of 5 ng/ml for IL11 and TGFβ1 and 100 nM for ANGII and antibodies at 2 µg/ml.

The efficacy of a neutralizing anti-IL11 antibody (X203) was tested in VSMCs using inhibition of MMP2 secretion, as used in other IL11-responsive cell types^15,16^. X203 inhibited both TGFβ1 and ANGII-induced MMP2 secretion from VSMCs (IC_50_ = 5.53 and 6.10 ng/ml respectively; Supplementary Fig. S9 online). In separate studies, X203 (2 µg/ml) also inhibited TGFβ1- and ANGII-induced proliferation (*P*_TGFβ1_ = 0.025, *P*_ANGII_ = 0.024), COL1A1 expression (*P*_TGFβ1_ < 0.001, *P*_ANGII_ = 0.021) and total collagen secretion (*P*_TGFβ1_ < 0.001, *P*_ANGII_ = 0.001; Fig. 2a,b). Additionally, X203 blocked TGFβ1- and ANGII-driven VSMC migration (*P*_TGFβ1_ = 0.025, *P*_ANGII_ = 0.006; Fig. 2c and Supplementary Fig. S10 online) as well as TGFβ1-stimulated VSMC invasion across the Matrigel barrier (*P* = 0.021; Fig. 2d). Taken together, these genetic and pharmacologic data show that IL11 is required for VSMC dedifferentiation downstream of TGFβ1 and ANGII.

### IL11 induces VSMC phenotypic switching via activation of the ERK pathway

Non-canonical ERK signaling, rather than STAT3 activation, appears the major mediator of the IL11-driven fibroblast-to-myofibroblast transformation^14^ and we assessed the activation of both pathways in IL11-stimulated VSMCs over a time course (Fig. 2e). STAT3 activation initially decreased (*P* < 0.001 at 1 h) after IL11 stimulation before returning to baseline levels at 24 h (Fig. 2e). In contrast, ERK signaling was rapidly induced by IL11 stimulation (*P* < 0.001 at 0.25 h) and remained elevated.

To complement the cellular assays that suggested IL11 is required for TGFβ1- or ANGII-induced phenotypic switching, we performed additional signaling studies (Fig. 2f). VSMCs stimulated with TGFβ1 or ANGII for 24 h in the presence of a control antibody exhibited increased ERK activation and elevated expression of COL3A1 and FN1, whereas VSMCs incubated with X203 did not.

To examine whether ERK signaling itself was specifically important for phenotypic switching, we used U0126 to block MEK/ERK activity and studied effects on IL11-induced VSMC dedifferentiation. U0126 inhibited IL11-induced VSMC proliferation and collagen expression (both *P* = 0.008; Fig. 2g).

Overall, these data show that IL11 signaling in VSMCs is driven via non-canonical ERK signaling, in accordance with our findings in other stromal cells^14^, and suggests that TGFβ1- and ANGII-induced VSMC phenotypic switching is, at least in part, IL11 dependent.

### SMC-specific IL11 expression causes aortic remodeling

To study the effects of IL11 on VSMCs in vivo, we crossed *Rosa26*^*Il11/*+^ transgenic mice^14^ with mice that express tamoxifen (TAM)-inducible Cre recombinase driven by the myosin heavy chain 11 (*Myh11*) promoter^29^. This model allows SMC-specific *Il11* overexpression in an inducible manner in adult mice (referred to here as *Il11*-Tg mice)^30^.

**Figure 3:**
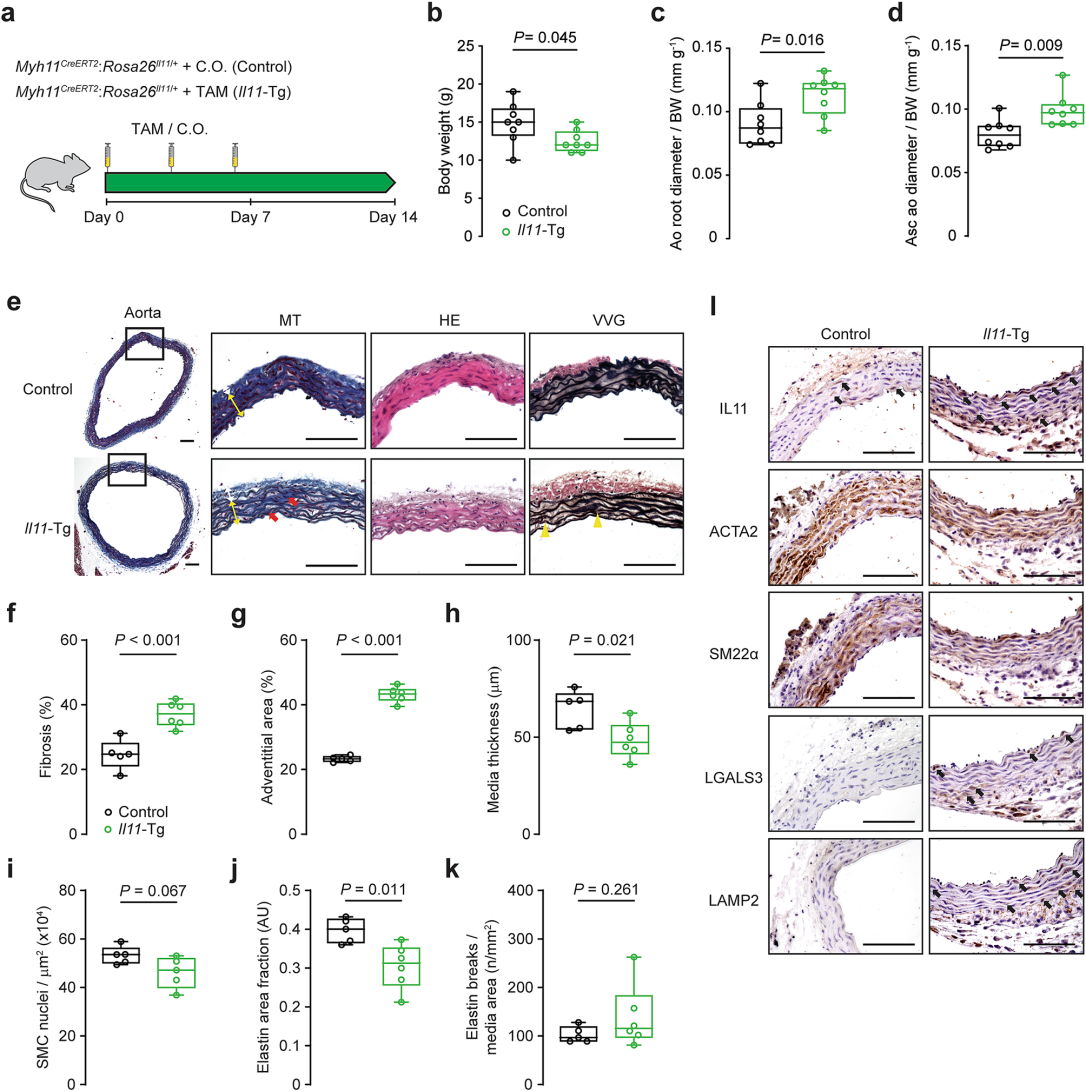
Smooth muscle cell-restricted IL11 expression induces aortic remodeling, fibrosis, and inflammation. (**a**) Schematic diagram featuring induction protocol of smooth muscle-specific *Il11* overexpression with 3 doses of 50 mg/kg tamoxifen (TAM) treatment in *Myh11*^*CreERT2*^:*Rosa26*^*IL11*/+^ (*Il11*-Tg) or corn oil vehicle-treated (control) littermates. Animals were sacrificed for studies at 8 weeks of age following a week of washout. (**b**) Body weights and aortic echocardiography for (**c**) aortic root diameter and (**d**) ascending aortic diameter normalized against individual body mass in *Il11*-Tg mice as compared with controls (*n* = 8/group). (**e**) Representative photomicrographs captured at 100X and 400X magnification of transverse aortic sections in *Il11*-Tg and control mice stained with Masson’s Trichrome (MT), hematoxylin & eosin (HE) and Verhoeff Van Gieson (VVG) stains. The white and yellow double-headed arrows demarcate the tunica adventitia and media layers respectively. Red arrows indicate media fibrosis and yellow arrowheads indicate lamellar elastin breaks. Histological analyses of (**f**) fibrosis, (**g**) adventitial area, (**h**) media thickness, (**i**) smooth muscle cell (SMC) nuclei, (**j**) elastin area and (**k**) elastic lamella breaks (*n* = 5–6 animals per group). Statistical analyses by unpaired t-test; data presented as median ± IQR, whiskers define the minimum and maximum values. (**l**) Representative photomicrographs (400X mag) of aortic sections in *Il11*-Tg and control mice immunostained for IL11, ACTA2, SM22α, LGALS3 and LAMP2 (*n* = 5/group). Black arrows indicate labeled VSMCs within the media. Scale bars represent 100 µm.

Following TAM induction, mice underwent an aortic ultrasound assessment followed by tissue collection for molecular characterization (Fig. 3a). *Il11-Tg* mice have previously been shown to have unchanging body weights upon TAM initiation, whereas vehicle-treated controls gain ~ 20% body mass between 6 and 8 weeks of age seen with normal growth in young mice^30^. At the study endpoint, body weights of *Il11*-Tg mice were reduced compared with their vehicle-treated littermates (*P* = 0.045; Fig. 3b). To account for the difference in body mass, aortic diameter measurements were normalized to individual body weights. Aortic ultrasound revealed an increase in normalized aortic root diameter and ascending aorta diameter in *Il11*-Tg mice as compared with controls (*P* = 0.016 and *P* = 0.009 respectively; Fig. 3c,d)—indicating spontaneous aortic remodeling associated with SMC-specific *IL11* overexpression.

Histological assessment of the SMC-specific *Il11* overexpressed aorta revealed increased vascular fibrosis (*P* < 0.001; Fig. 3e,f), predominantly in the fibroblast-rich adventitial region (*P* < 0.001; Fig. 3g) that suggests paracrine effects. Smooth muscle thickness and VSMC numbers were mildly reduced in *Il11*-Tg aortas (*P* = 0.021 and *P* = 0.067 respectively; Fig. 3h,i), suggestive of medial VSMC degeneration. Elastin area, but not elastic break counts, was significantly reduced in *Il11*-Tg aortas (*P* = 0.011; Fig. 3j,k) although this may reflect the short induction period. IL11 protein expression was scant in controls but upregulated in both adventitial fibroblasts and VSMCs in *Il11*-Tg aortas (Fig. 3l). In contrast, ACTA2 and transgelin (SM22α) expression was reduced in the VSMCs of *Il11*-Tg aortas. To investigate the impact of IL11 expression on aortic inflammation, we probed for galectin-3 (LGALS3) and lysosome-associated membrane protein-2 (LAMP2) expression which are known markers for macrophages but also rarely expressed in VSMCs in response to atherosclerosis or injury^31–33^. Not only did LGALS3 and LAMP2 expression increase in macrophages of the tunica intima and adventitia of *Il11*-Tg aortas (Fig. 3l), but the VSMC-rich media also demonstrated elevated expression which suggests intrinsic VSMC inflammation, albeit at a much lower intensity compared to macrophages in the adventitia.

**Figure 4:**
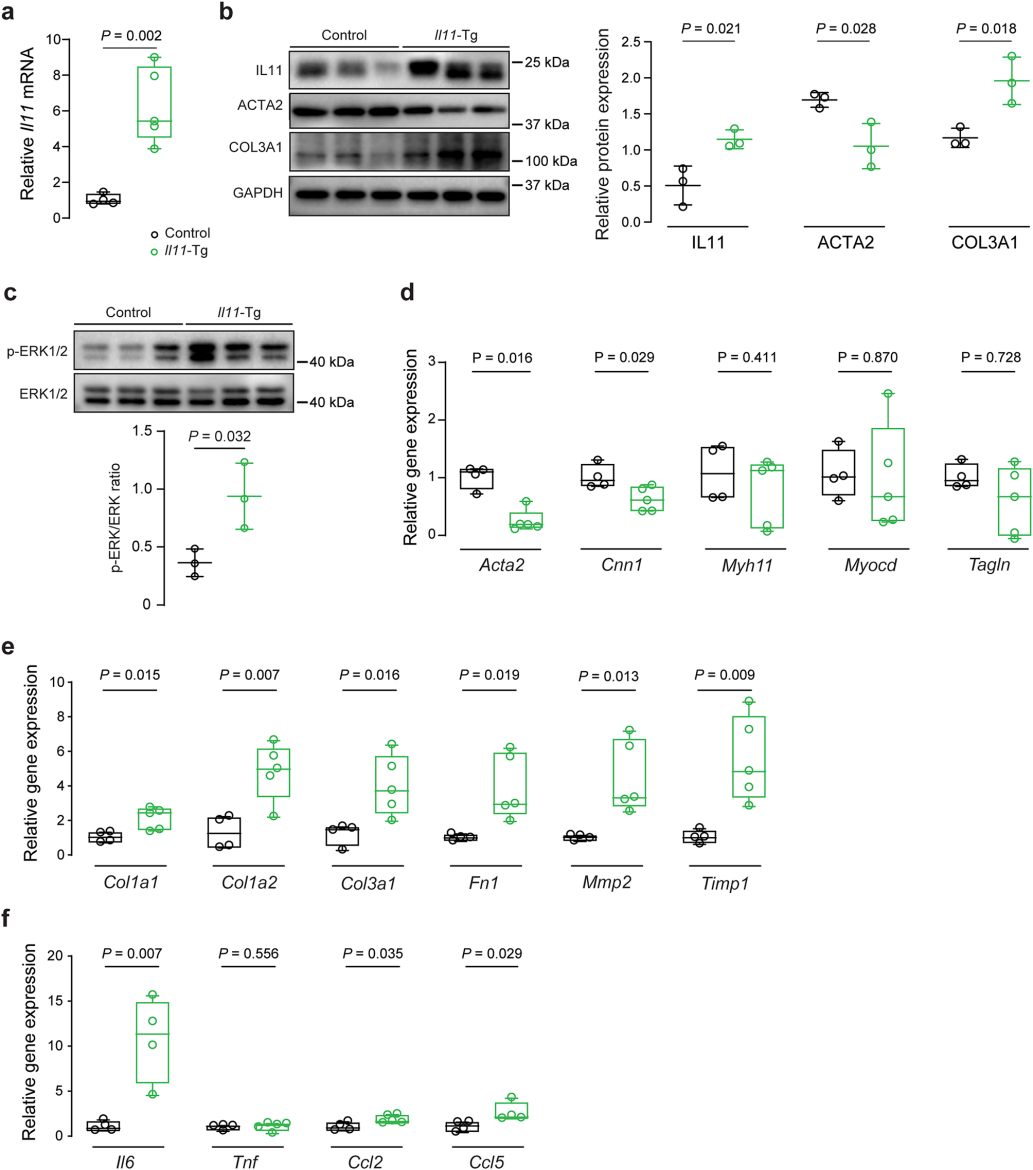
Smooth muscle cell-restricted IL11 expression induces molecular changes of aortic remodeling. (**a**) RT-qPCR of *Il11* mRNA expression normalized to *Gapdh* expression in aortas of *Il11*-Tg and control mice (n = 5/group). Representative immunoblots for aortic expression of (**b**) IL11, ACTA2, COL3A1 and GAPDH protein and (**c**) phosphorylated (p) and total ERK1/2 protein expression in *Il11*-Tg mice as compared with controls (*n* = 3/group). Collated densitometry for IL11, ACTA2 and COL3A1 normalized to GAPDH and p-ERK1/2 was normalized to total ERK1/2 protein expression. Samples were derived from the same experiment and blots were processed in parallel. Extended blots are presented in Supplementary Fig. S13 online. Data expressed in mean ± SD. RT-qPCR of (**d**) contractile genes (Acta2*, *Cnn1*, *Myh11*, *Myocd*, and *Tagln*), (**e**) ECM genes (*Col1a1*, *Col1a2*, Col3a1*, *Fn1*, *Mmp2*, and *Timp1*), and (**f**) inflammatory genes (*Il6*, Tnf*, *Ccl2* and Ccl5*) normalized to *Gapdh* expression in aortas of *Il11*-Tg and control mice (*n* = 4–5/group). Statistical analyses by unpaired t-test unless data deviated from normal, in which case a Mann-Whitey test was performed (denoted by *). Data presented as median ± IQR, whiskers define the minimum and maximum values.

RT-qPCR of *IL11* mRNA and immunoblots of the protein in thoracic aorta homogenates demonstrated elevated IL11 expression in *Il11*-Tg mice, confirming the expression of the transgene (*P* = 0.002 and *P* = 0.021 respectively; Fig. 4a,b). *Il11*-Tg aortas also had elevated collagen 3 (COL3A1) levels with concurrent reduction in alpha smooth muscle actin (ACTA2) (*P*_COL3A1_ = 0.018, *P*_ACTA2_ = 0.028; Fig. 4b). Additionally, ERK signaling was increased in the aortas of *Il11*-Tg mice (*P* = 0.032, Fig. 4c).

We next examined contractile, ECM and inflammatory gene expression in the mouse aortas. *Il11*-Tg mice had reduced *Acta2* (*P* = 0.016) and calponin-1 (*Cnn1)* (*P* = 0.029) expression whereas *Myh11*, myocardin (*Myocd)* and transgelin (*Tagln*) were unchanged (Fig. 4d). In contrast, gene expression of ECM components were elevated (*P*_*Col1a1*_ = 0.015, *P*_*Col1a2*_ = 0.007, *P*_*Col3a1*_ = 0.016, *P*_*Fn1*_ = 0.019, *P*_*Mmp2*_ = 0.013, *P*_*Timp1*_ = 0.009; Fig. 4e) and inflammatory markers, of which *Il6* was induced most strongly ~ tenfold (*P*_*Il6*_ = 0.007, *P*_*Ccl2*_ = 0.035, *P*_*Ccl5*_ = 0.029; Fig. 4f). Overall, these in vivo gain-of-function data show that secretion of IL11 from VSMCs is sufficient to cause aortic remodeling, fibrosis and inflammation.

### IL11 antibodies reduce aortic constriction-induced aortic remodeling

**Figure 5:**
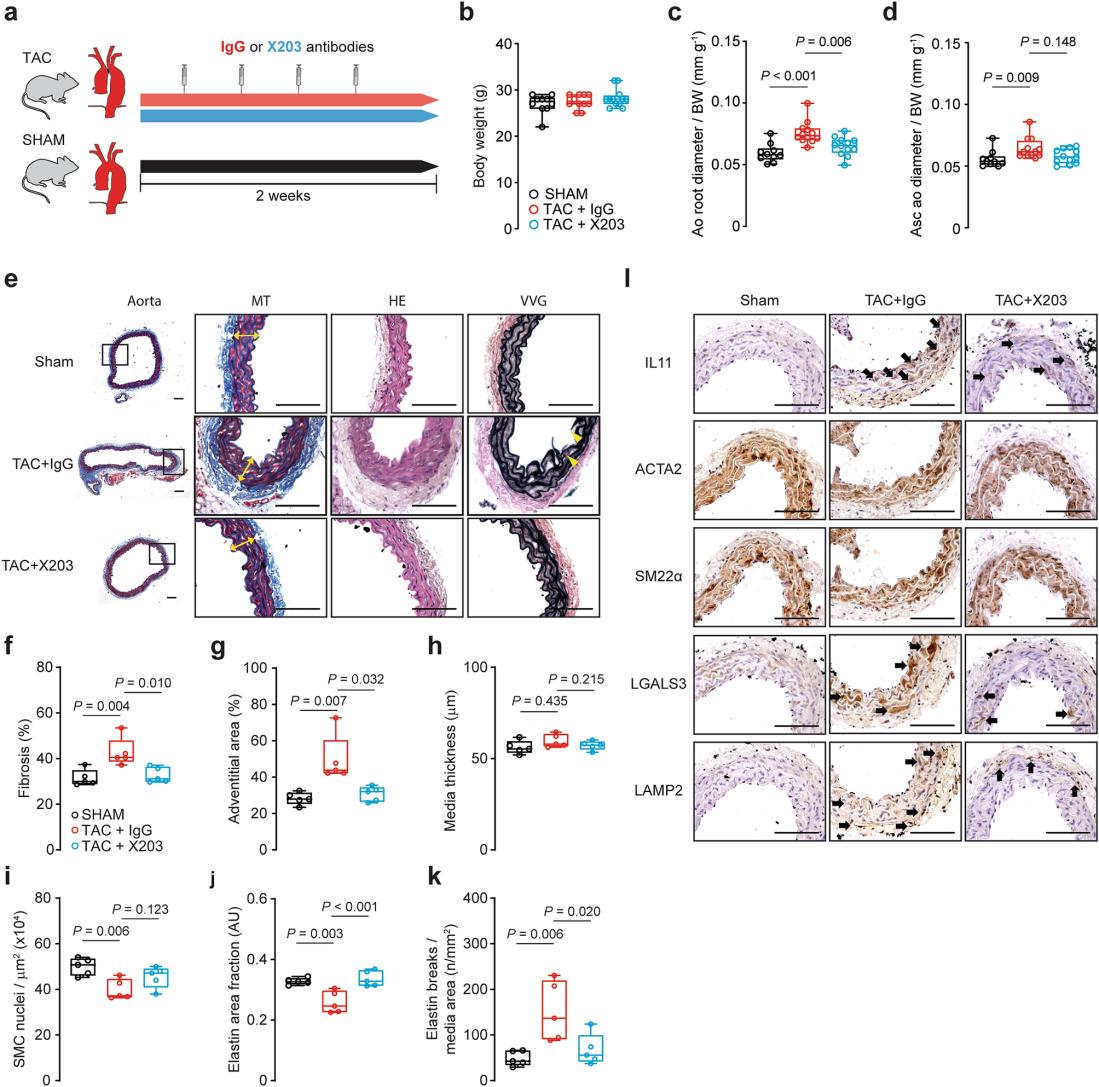
Antibody-mediated inhibition of IL11 reduces aortic constriction-induced aortic remodeling. (**a**) Schematic diagram depicting TAC experimental protocol. Wildtype C57BL/6 J mice were injected with X203 or IgG control antibodies (20 mg/kg IP twice per week) starting from 24 h post-TAC surgery. (**b**) Body weights* and aortic echocardiography for (**c**) aortic root diameter and (**d**) ascending aortic diameter* in Sham, TAC + IgG or TAC + X203 treated animals (*n* = 10–12/group). Aortic diameters were normalized to individual body mass. (**e**) Representative photomicrographs captured at 100X and 400X magnification of transverse aortic sections in sham, TAC + IgG, and TAC + X203 mice stained with Masson’s Trichrome (MT), hematoxylin and eosin (HE) and Verhoeff Van Gieson (VVG) stains. The white and yellow double-headed arrows demarcate the tunica adventitia and media layers respectively. Yellow arrowheads indicate lamellar elastin breaks. Histological analyses of (**f**) fibrosis, (**g**) adventitial area*, (**h**) media thickness, (**i**) SMC nuclei, (**j**) elastin area and (**k**) elastic lamella breaks (*n* = 5/group). Statistical analyses by one-way ANOVA with Sidak multiple comparisons, unless data deviated significantly from normal, in which case a Kruskal–Wallis test with Dunn’s multiple comparisons was performed (denoted by *). Data presented as median ± IQR, whiskers define the minimum and maximum values. (**l**) Representative photomicrographs captured at 400X magnification of transverse aortic sections in sham, TAC + IgG, and TAC + X203 mice immunostained with anti-IL11, ACTA2, SM22α, LGALS3, and LAMP2 (*n* = 3/group). Black arrows indicate labeled VSMCs within the media. Scale bars represent 100 µm.

We next tested the effect of pharmacologic inhibition of IL11 signaling using the IL11 antibody (X203) in the transverse-aortic constriction (TAC)^4^ model of aortic remodeling (Fig. 5a). 2-weeks post-TAC there was no difference in body weights between groups (Fig. 5b). X203 administration significantly reduced aortic root size by ~ 33% (*P* = 0.006, Fig. 5c) despite equal TAC-induced pressure overload as assessed by echocardiographic pressure gradient (mean ± SD; IgG vs. X203: 77.0 ± 9.1 vs. 75.5 ± 10.0 mmHg; *P* = 0.7). The ascending aorta diameter also tended to be smaller in X203 treated mice but this did not reach significance (*P* = 0.148, Fig. 5d).

Histological assessment of X203 effects in TAC-treated aorta revealed reduced vascular fibrosis (*P* = 0.010; Fig. 5e,f), predominantly in the fibroblast-rich adventitial region (*P* < 0.001; Fig. 5g). Whilst we did not observe significant changes in TAC-induced media thickness (Fig. 5h), reduction in SMC nuclei showed a trend towards improvement with X203 treatment although this did not reach significance (*P* = 0.123; Fig. 5i). X203 significantly prevented both elastin area and break counts in TAC-treated aortas (*P* < 0.001 and *P* = 0.02 respectively; Fig. 5j,k). TAC-induced increased IL11 protein expression, in both the adventitia and media, was inhibited by X203 (Fig. 5l). ACTA2 and SM22α staining was mildly reduced with TAC without apparent effect of X203, whereas TAC-induced elevated LGALS3 and LAMP2 expression in the tunica media was reduced with X203 treatment.

**Figure 6:**
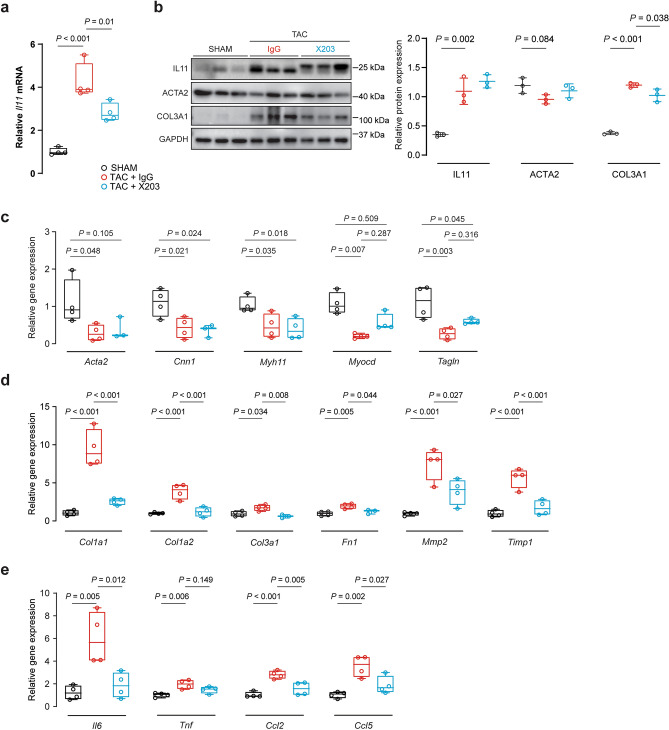
Anti-IL11 antibodies reduce TAC-induced molecular changes in the mouse aorta. (**a**) RT-qPCR of *Il11* mRNA expression normalized to *Gapdh* expression in aortas of Sham, TAC + IgG or TAC + X203 mice (*n* = 4/group). (**b**) Representative immunoblots of IL11, ACTA2, COL3A1, and GAPDH in the aorta of Sham, TAC + IgG or TAC + X203 treated animals (n = 3/group). Collated densitometry for IL11, ACTA2 and COL3A1 normalized to GAPDH protein expression. Samples were derived from the same experiment and blots were processed in parallel. Extended blots are presented in Supplementary Fig. S14 online. Data expressed in mean ± SD. RT-qPCR of (**c**) contractile genes (*Acta2*, *Cnn1*, *Myh11*, Myocd*, and *Tagln*), (**d**) ECM genes (*Col1a1*, *Col1a2*, *Col3a1*, *Fn1*, *Mmp2*, and *Timp1*), and (**e**) inflammatory genes (*Il6*, *Tnf*, *Ccl2* and *Ccl5*) normalized to *Gapdh* expression in Sham, TAC + IgG or TAC + X203 mice (*n* = 3–4/group). Statistical analyses by one-way ANOVA with Sidak multiple comparisons unless data deviated from normal, in which case a Kruskal–Wallis test was performed (denoted by *). Data presented as median ± IQR, whiskers demarcate the minimum and maximum values.

Aortic tissue homogenates showed increased mRNA and protein expression of IL11 with TAC (*P* < 0.001 and *P* = 0.002 respectively; Fig. 6a,b), which was partially reduced with X203 treatment at the gene but not the protein level, differing somewhat from the histological profile. Immunoblots showed that the molecular weight of IL11 protein shifted slightly with X203 treatment, as we have seen previously^16^, which may represent target engagement or unrelated post-translational modifications. While ACTA2 expression was not significantly different after antibody treatment, X203 significantly reduced COL3A1 expression (*P* = 0.038, Fig. 6b).

At the RNA level, X203 treatment did not restore TAC-induced reduction in contractile genes (Fig. 6c). In contrast, ECM genes were significantly reduced with X203 (*Col1a1*, *Col1a2*, *Col3a1*, *Fn1*, *Mmp2* and *Timp1*, all *P* < 0.05, Fig. 6d). Similarly, expression of the inflammatory genes *Il6*, *Ccl2* and *Ccl5* (but not *Tnf*) were significantly reduced following X203 treatment (*P*_*Tnf*_ = 0.149, all others *P* ≤ 0.05, Fig. 6e).

### IL11 antibodies reduce angiotensin II-induced aortic remodeling

**Figure 7:**
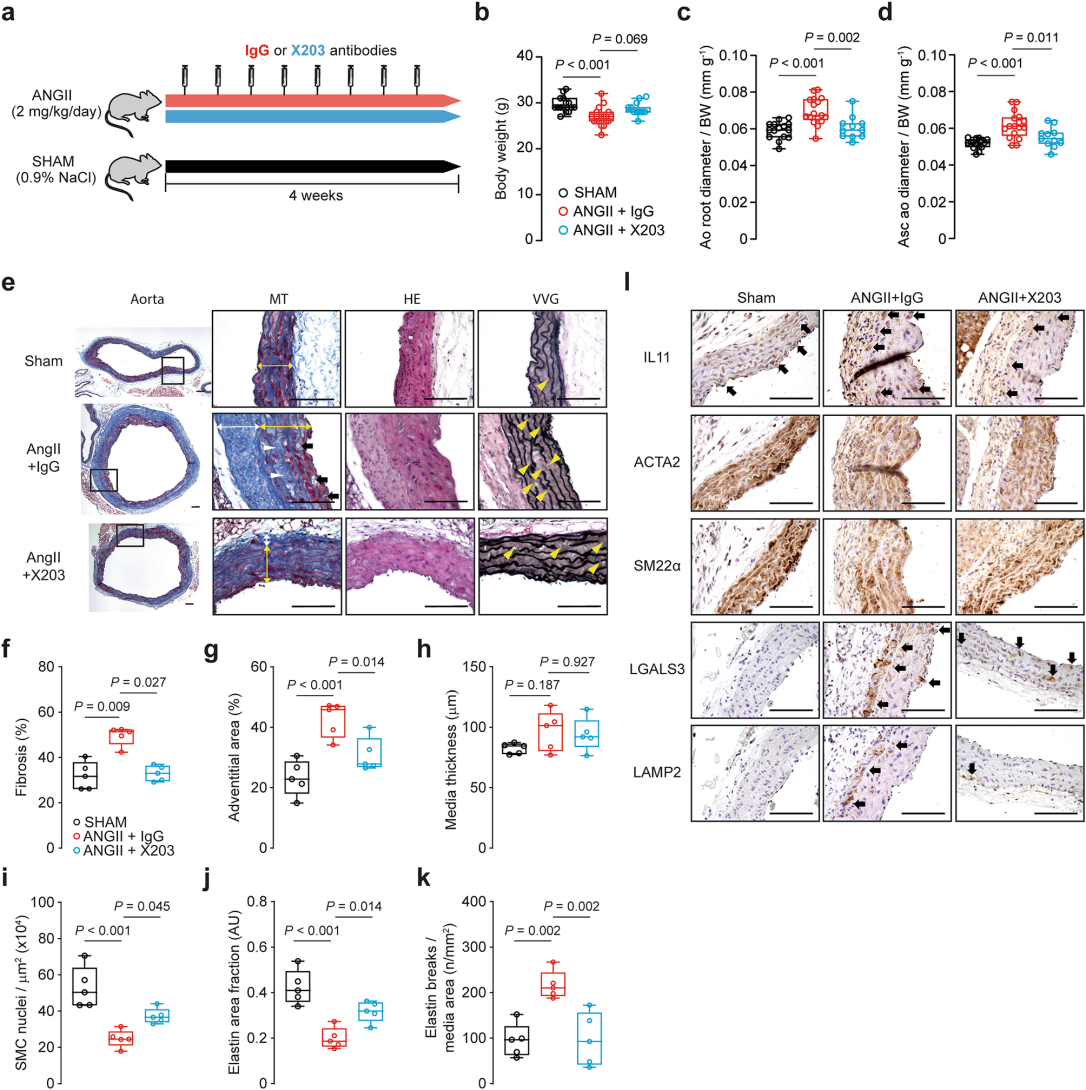
Administration of IL11 antibodies prevents ANGII-induced aortic remodeling. (**a**) Schematic diagram depicting ANGII experimental protocol. Wildtype C57BL/6 J mice were injected with X203 or IgG control antibodies (20 mg/kg via IP twice per week) starting from 24 h after being implanted with osmotic minipumps containing ANGII. (**b**) Body weights and aortic echocardiography for (**c**) aortic root diameter and (**d**) ascending aortic diameter in Sham, ANGII + IgG, ANGII + X203 animals (*n* = 11–17/group). (**e**) Representative photomicrographs captured at 100X and 400X magnification of transverse aortic sections in sham, ANGII + IgG, and ANGII + X203 mice stained with Masson’s Trichrome (MT), hematoxylin and eosin (HE) and Verhoeff Van Gieson (VVG) stains. The white and yellow double-headed arrow demarcates the tunica adventitia and media layers respectively. Black arrows indicate cell-free foci of proteoglycan-rich matrix, white and yellow arrowheads indicate replacement fibrosis in the tunica media and lamellar elastin breaks respectively. Histological analyses of (**f**) fibrosis*, (**g**) adventitial area, (**h**) media thickness, (**i**) SMC nuclei, (**j**) elastin area and (**k**) elastic lamella breaks (*n* = 5/group). Statistical analyses by one-way ANOVA with Sidak multiple comparisons, unless data deviated significantly from normal, in which case a Kruskal–Wallis test with Dunn’s multiple comparisons was performed (denoted by *). Data presented as median ± IQR, whiskers define the minimum and maximum values. (**l**) Representative photomicrographs captured at 400X magnification of transverse aortic sections in sham, ANGII + IgG, and ANGII + X203 mice immunostained with anti-IL11, ACTA2, SM22α, LGALS3, and LAMP2 (*n* = 3/group). Black arrows indicate labeled VSMCs within the media. Scale bars represent 100 µm.

To test further the effects of IL11 antibodies on aortic remodeling, X203 was administered in a second model of pressure overload using ANGII infusion^34,35^ (Fig. 7a). We have previously demonstrated no effect of IL11 loss-of-function on blood pressure at baseline (over 7 days) or ANGII-induced hypertension (over 28 days) in mice using telemetry^14^. There was mild but significant weight loss induced by ANGII (*P* < 0.001; Fig. 7b). As in the TAC model, X203 administration significantly inhibited ANGII-induced aortic root size (15%, *P* = 0.002) and dilatation of the ascending aorta (35%, *P* = 0.011) (Fig. 7c,d).

Histological assessment of the X203 treatment in ANGII-treated aorta revealed reduced vascular fibrosis (*P* = 0.027; Fig. 7e,f), both in the adventitia (*P* < 0.001; Fig. 7g) as well as replacement fibrosis in the tunica media (Fig. 7e). In our model, 28 days of ANGII induction did not alter media thickness (Fig. 7h). For other phenotypes, X203 significantly restored SMC nuclei (*P* = 0.045; Fig. 7i) and prevented elastin area and break counts in ANGII-treated aortas (*P* = 0.014 and *P* = 0.002 respectively; Fig. 7j,k). ANGII-treated aortas had elevated IL11 protein expression, reduced SMC ACTA2 and SM22α expression, and elevated immune cell markers LGALS3 and LAMP2 (Fig. 7l). X203 treatment attenuated these protein expression changes in the aorta.

**Figure 8:**
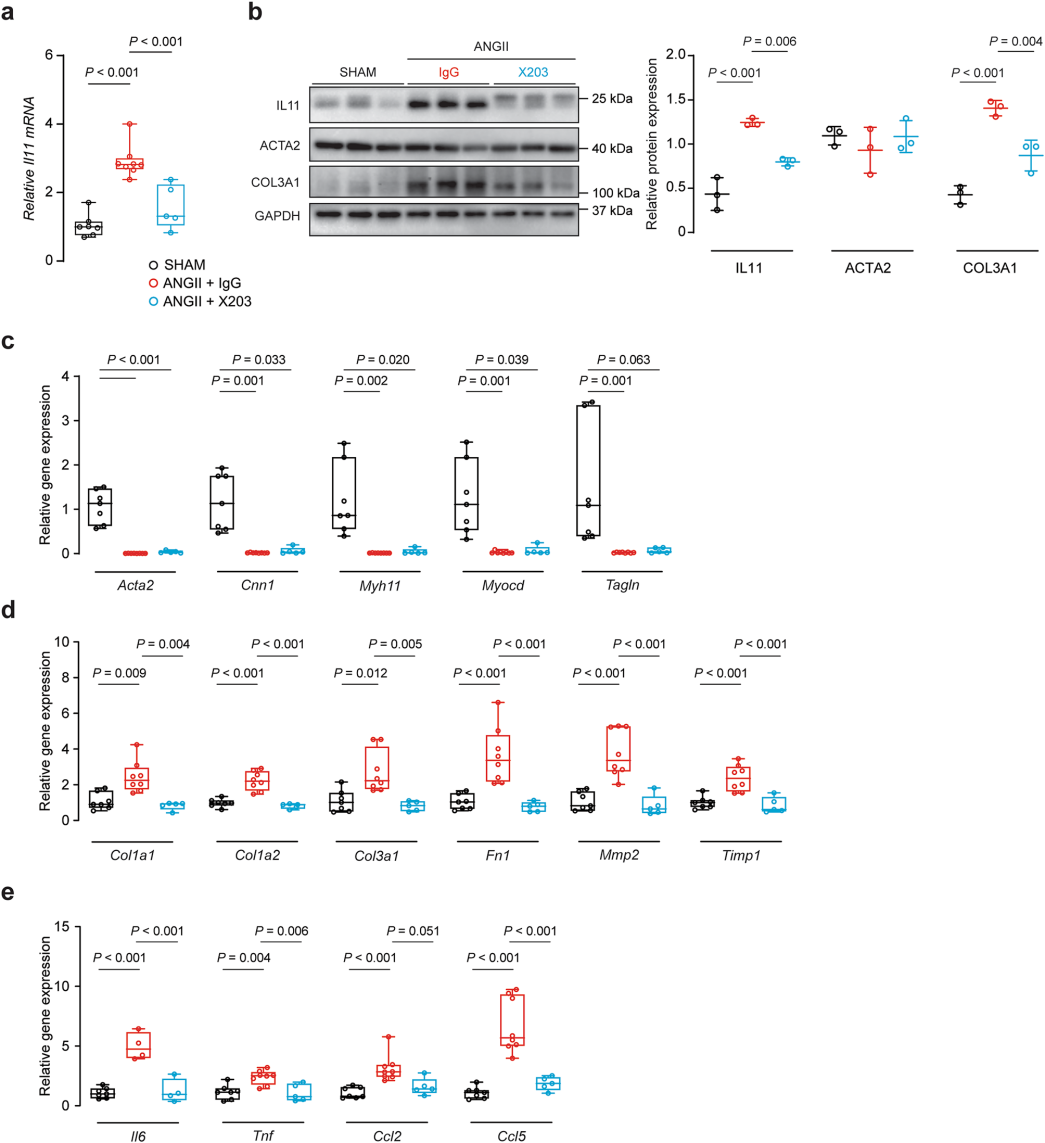
Anti-IL11 treatment prevents ANGII-induced histological changes in the mouse aorta. (**a**) RT-qPCR of *Il11* gene expression normalized to *Gapdh* expression (*n* = 5–8/group). (**b**) Representative immunoblots of IL11, ACTA2, COL3A1, and GAPDH in the aortas from Sham, ANGII + IgG or ANGII + X203 animals (*n* = 3/group). Collated densitometry for IL11, ACTA2 and COL3A1 normalized to GAPDH protein expression. Samples were derived from the same experiment and blots were processed in parallel. Extended blots are presented in Supplementary Fig. S14 online. Data expressed in mean ± SD. RT-qPCR of (**c**) contractile genes (*Acta2*, Cnn1*, Myh11*, Myocd*, and Tagln*), (**d**) ECM genes (Col1a1*, *Col1a2*, Col3a1*, *Fn1*, *Mmp2*, and *Timp1*), and (**e**) inflammatory genes (*Il6*, *Tnf*, Ccl2* and *Ccl5*) normalized to *Gapdh* expression in the aortas of ANGII mice treated with IgG or X203 antibodies compared with sham controls (*n* = 4–8/group). Statistical analyses by one-way ANOVA with Sidak multiple comparisons, unless data deviated significantly from normal, in which case a Kruskal–Wallis test with Dunn’s multiple comparisons was performed (denoted by *). Data presented as median ± IQR, whiskers demarcate the minimum and maximum values.

IL11 mRNA was induced by ANGII which was inhibited by X203 (*P* < 0.001, Fig. 8a). By western blot, X203 inhibited ANGII-induced IL11 and COL3A1 protein expression whereas ACTA2 levels were unaffected (*P*_*IL11*_ < 0.001, *P*_COL3A1_ = 0.004, Fig. 8b). At the RNA level, X203 did not restore ANGII-induced decrease in contractile gene transcription (Fig. 8c) but prevented ANGII-induced expression of ECM (*Col1a1*, *Col1a2*, *Col3a1*, *Fn1*, *Mmp2* and *Timp1*, all *P* ≤ 0.01, Fig. 8d) and inflammatory genes (*Il6, Tnf and Ccl5*, all *P* ≤ 0.01, Fig. 8e) with a trend towards decrease in *Ccl2* (*P* = 0.051).

## Discussion

IL11 is a key downstream mediator of TGFβ1 and ANGII effects in cardiac fibroblasts^14^, lung fibroblasts^16^ and hepatic stellate cells^15^, where it is required for ERK-dependent myofibroblast activation. TGFβ1 has been shown to induce IL11 secretion from aortic and coronary artery VSMCs^17,19^. This finding, which we confirm, and the fact that VSMCs highly express IL11RA, which we demonstrate, suggest the existence of an autocrine loop of IL11 activity in VSMCs.

While IL11 is often thought of in the same context as its family member IL6, our data show that these two cytokines have different roles in VSMCs. *IL6R* RNA levels are much lower than *IL11RA* in VSMCs and while IL11RA protein expression is abundant, IL6R is not readily detectable. This is consistent with data showing that VSMCs are unresponsive to IL6 stimulation directly^23^, although some studies suggest otherwise^36^. It has been suggested that soluble IL6R (sIL6R), through shedding or alternative splicing mechanisms, forms an activation loop to drive pro-inflammatory VSMC transition^23^. Based on our data, the source of sIL6R is unlikely to be VSMCs themselves, but neighboring cell types could shed this protein. Pro-inflammatory cytokines induce both IL11 and IL6 secretion from VSMCs, but we suggest that their subsequent modes of action are different: IL11 has autocrine activity in VSMCs and likely paracrine activity on other cells that carry IL11RA, such as fibroblasts. On the other hand, IL6 in itself has little direct impact on VSMCs, and instead exerts paracrine effects on local and circulating immune cells^17,19,36^.

The TGFβ1 pathway is central to syndromic thoracic aortic aneurysm and dissection, typified by Marfan syndrome^37–39^. In mouse models of Marfan, aortic aneurysm development can be reversed by pan-specific TGFβ neutralizing antibodies, angiotensin II receptor blockers or inhibiting ERK signaling^9,10,40^. However, targeting TGFβ1 is associated with dose-limiting toxicities and aortic dissection^41–44^. We show that IL11 is required downstream of TGFβ1 and ANGII specifically for ERK-dependent VSMC phenotypic switching and it is therefore possible that IL11 signaling could be involved in the pathogenesis of Marfan aortopathy.

There is accumulating evidence that ANGII and TGFβ signaling are intricately linked in VSMCs, sharing similar canonical and noncanonical pathways in phenotype switching^12,21^. Indeed, TAC-induced TGFβ-dependent aortic remodeling can be attenuated with ANGII receptor blockers^4^. Our findings place IL11 downstream of both ANGII and TGFβ signaling pathways and show that IL11 directly induces VSMC phenotypic switching. This discovery uncovers a novel biological function of IL11 in the vasculature.

It is important to highlight that IL11 was previously thought to be anti-inflammatory in the cardiovascular system^45–47^ but we show its expression induces a pro-inflammatory response when expressed in VSMCs (Fig. 3). Consistent with a pro-inflammatory role for IL11, histological assessment of the aorta revealed elevated LGALS3 and LAMP2, common macrophage markers, in the smooth muscle-rich tunica media which was reduced with anti-IL11 treatment (Figs. 5 and 7). Furthermore, anti-IL11 antibodies not only reduce aortic remodeling and pro-fibrotic gene expression but also pro-inflammatory gene expression, notably IL6 (Figs. 6 and 8). Expression of IL11RA on parenchymal and stromal cells, but not immune cells^14^, suggests that the beneficial effects on inflammation associated with IL11 inhibition are indirect, as seen recently by proinflammatory fibroblasts^48^.

In human disease, IL11 levels are elevated in the serum and aortic tissue of patients with thoracic aortic dissections^49^. Similarly, serum IL11 was found to be raised in patients with coronary atherosclerosis and unstable angina, which might reflect its secretion from dysfunctional coronary artery VSMCs^19,50^. A possible link of IL11 with coronary artery disease is intriguing given the central role of VSMC phenotypic switching in atherosclerosis and the fact that anti-inflammatory therapies are proving effective in patients with coronary disease^51,52^.

Our study has limitations. To achieve sufficient cell numbers for our experimental studies we used VSMCs of early passages (≤ P4) that while an accepted approach is not as close to the in vivo state as freshly dispersed VSMCs^53–56^. Another limitation was the mortality in SMC-overexpressing IL11 transgenic mice driven, in part, by inflammatory bowel disease^30^. Nonetheless, our genetic and pharmacological loss-of-function experiments showed modulation of VSMC and aortic pathobiology in the absence of systemic phenotypes. We did not observe aortic dissection in our ANGII model, that has been described in some studies^8,35^, and only non-dissected aortas were analyzed. Vascular fibrosis was quantified as the percentage of total fibrosis area which closely mirrors adventitial fibrosis. Medial fibrosis quantification was not individually measured due to a lack of medial fibrosis in our TAC model. Whilst we did not measure blood pressure in our ANGII model, the TAC model compensates for this and we have shown previously that inhibition of IL11 signaling in mice has no effect on ANGII-induced hypertension^14^.

IL11 is a little-studied cytokine, particularly in the context of vasculature. We show here that IL11 is required for VSMC phenotypic switching. IL11 levels are elevated in human aortic disease^49^ and we found that IL11 is important for aortic remodeling in mouse models of aortopathy, where VSMC phenotypic switching is important^5,6,8^. Hence, inhibition of IL11 signaling, which is predicted to have a favorable safety profile^57^, may be beneficial in aortic disease. IL11 was recently found to be increased in pulmonary arterial VSMCs from patients with pulmonary arterial hypertension^58^ and IL11 may be more generally important for diseases characterized by VSMC dysfunction, which requires further study.

## Methods

Expanded methods are available online in the Supplementary Methods section. All reagents and methods pertaining to immunofluorescence and confocal microscopy, flow cytometry, migration and invasion assays, enzyme-linked immunoassay, collagen assays, immunoblotting and RT-qPCR are also provided in the Supplementary Information online.

### Ethics statements

All experimental protocols involving human subjects were approved by the SingHealth Centralized Institutional Review Board (2013/103/C and 2018/2543) in accordance with the *ICH Guidelines for Good Clinical Practice* and all participants gave written informed consent.

Animal studies were carried out in compliance with the recommendations in the *Guidelines on the Care and Use of Animals for Scientific Purposes* of the *National Advisory Committee for Laboratory Animal Research* (NACLAR). All experimental procedures were approved (SHS/2014/0925) and conducted in accordance with the SingHealth Institutional Animal Care and Use Committee.

### Human VSMCs cell culture

VSMCs were maintained in complete M231 medium (M-231-500) with smooth muscle growth supplement (S-007-25) and 1% antibiotic–antimycotic (15240062) from Life Technologies, in a humidified atmosphere at 37 °C and 95% air/5% CO_2_. At passage 1–2, VSMCs were negatively selected by magnetic separation for CD90 and CD144 (130-096-253 and 130-097-857 respectively, Miltenyi Biotec) for experiments.

Experiments were carried out at low cell passages (≤ passage 4) and cells were growth restricted with 0.2% fetal bovine serum (FBS) in basal M231 for 24 h before IL11 (5 ng/ml), TGFβ1 (5 ng/ml), or ANGII (100 nM) treatment in serum-free M231 for 24 h. Stimulated VSMCs were compared to unstimulated VSMCs for the same duration under basal M231 medium only. For MEK/ERK inhibition studies, VSMCs were stimulated with IL11 in the presence of U0126 (10 µM) reconstituted in dimethyl sulfoxide (DMSO) compared to vehicle controls.

### RNA-sequencing

Bulk RNA-seq analyses was performed as previously described^14,59,60^. For GO over-representation analysis, differentially expressed genes (FDR < 0.05) were tested for statistical significance against the background of all expressed genes using gProfileR^61^ with “strong” hierarchical filtering. Gene Set Enrichment Analyses (GSEA) were run using the fgsea library^62^, pre-ranking the gene list by the “stat” column of the DESeq2 results output, and using 10^5^ permutations.

### FANTOM5 data processing

Gene expression data in primary cell types with replicates were downloaded from the FANTOM5 web resource^22^. Gene level expression was calculated by summing all counts followed by normalization of library size to calculate the tags per million (TPM) for IL11RA and IL6R.

### Mouse models

All mice were from the C57BL/6 genetic background and were housed under ABSL-1 conditions in the SingHealth Experimental Medicine Centre and provided normal chow and water ad libitum. Animals were euthanized at endpoint by ketamine (100 mg/kg) and xylazine (10 mg/kg) given IP, followed by the removal of vital organs and tissues. For surgeries, animals were anesthetized with 2–3% isoflurane and ventilated with a rodent ventilator (MiniVent Model 845, Hugo Sachs Elektronik). Postoperative analgesia and antibiotics, buprenorphine (0.1 mg/kg SQ; twice a day) and enrofloxacin (5 mg/kg SQ) respectively, were given daily up to 3 days post-surgery.

#### IL11 receptor null primary VSMCs

Four-to-six week old mice lacking functional alleles for *Il11ra1* (*Il11ra1*^−/−^, KO) and wild-type littermates (*Il11ra1*^+/+^, wildtype) were used for aortic VSMC extraction and culture using a modified protocol adapted from published literature^63,64^. At passage 2, VSMCs were negatively selected for CD45 (leukocytes), CD90.2 (fibroblasts), and CD31 (endothelial cells).

Passage 3 to 4 mouse aortic VSMCs were used for scratch wound migration assay or for collagen secretion assay. Murine VSMCs were either treated with recombinant mouse TGFβ1 (5 ng/ml), IL11 (5 ng/ml), or ANGII (100 nM) in M231 basal medium.

#### Smooth-muscle specific IL11 overexpressing mice

Heterozygous *Rosa26-IL11* (C57BL/6 N-*Gt(ROSA)26Sor*^*tm1(CAG-Il11)Cook*^/J) mice^14^ were crossed to the hemizygous *Myh11-CreERT2* (B6.FVB-Tg(Myh11-cre/ERT2)1Soff/J) mice^29^ (Jackson Laboratory; 031928 and 019079 respectively) to generate double heterozygous *Myh11*^*CreERT2*^*:Rosa26*^*IL11/*+^ offspring. *Myh11*^*CreERT2*^*:Rosa26*^*IL11/*+^ mice (*n* = 33) were injected with 3 doses of 50 mg/kg TAM (T5648, Sigma Aldrich) IP at 6 weeks of age to induce Cre-mediated *Il11* transgene induction. Control littermates were injected with equal volumes of corn oil (C8267, Sigma Aldrich). Mice were euthanized at 14 days from the first TAM dose.

#### Transverse aortic constriction (TAC)

TAC surgeries were performed on C57BL/6 J male mice (*n* = 24) as described^65^. Age-matched sham controls underwent the same operative procedure without ligation (*n* = 10). Trans-thoracic two-dimensional Doppler echocardiography was used to confirm increased pressure gradients (> 40 mmHg) indicative of successful TAC. Mice were randomized to receive post-operative antibody treatment conducted by IP injections of either X203 or IgG control antibodies (*n* = 12 per group) at a dose of 20 mg/kg twice per week for two consecutive weeks starting 24 h following TAC. Mice were euthanized at 2 weeks post-TAC prior to maladaptive heart failure response^65^ and the proximal ascending aorta was excised for molecular assessments.

#### Angiotensin II (ANGII) infusion

ANGII pump infusions were performed as previously described^14^. C57BL/6 J male mice were implanted with an osmotic minipump (Alzet model 1004, Durect) containing either angiotensin II (ANGII, 2 mg/kg/day SQ; *n* = 28) or an identical volume of saline. ANGII-treated mice were randomized to receive post-operatively injections of either X203 or IgG control antibodies at a dose of 20 mg/kg twice per week for four consecutive weeks. Mice underwent aortic echocardiography and were euthanized at 4 weeks post-ANGII and the thoracic aorta was excised for studies. No mortality associated with ANGII infusion was observed.

### Aortic echocardiography

In vivo trans-thoracic echocardiography was conducted using Vevo 2100 with a MS400 linear array transducer (VisualSonics), 18–38 MHz by a trained echocardiographer (NGZT) blinded to genotype and treatment groups. Aortic root and ascending aortic diameters were assessed from *B-* and *M*-mode of parasternal long-axis view, using inner edge-to-inner edge according to established guidelines^66^. Peak aortic flow velocity was obtained by applying pulsed-wave Doppler across the aortic valve from the aortic arch at suprasternal view. All measurements were averaged over three cardiac cycles. Aortic dimensions were referenced to body weight per animal to account for differences in body mass.

### Histology

Transverse sections (5 µm) of paraffin-embedded proximal ascending aorta were used for histological stains with Masson’s Trichrome (HT15, Sigma-Aldrich) for collagen, hematoxylin and eosin (H&E) for nuclei, and Verhoeff Van Gieson (VVG; 87017, Thermo Fisher) for elastin. Brightfield photomicrographs were randomly captured by a researcher (XYK) blinded to the treatment groups using the Olympus BX51 microscope and Image-Pro Premier 9.2 (Media Cybernetics).

Total fibrosis was measured by ImageJ (v1.52a, NIH) with *Colour Deconvolution*-Masson’s Trichrome expressed as a percentage of collagen stained area over total tissue area. Media thickness was measured as the intima-media distance using the incremental distance tool at a calibrated step of 10 μm on Image-Pro Premier 9.2 (Media Cybernetics) and reported as an average of 62–142 measurements per section.

Adventitial area and SMC nuclei were quantified in H&E stained sections as described^67^. Adventitial area was expressed as a percentage of total tissue area. SMC nuclei were expressed as nuclei counts over media area. Elastin area fraction was expressed as the elastin area over total tissue area. Elastin breaks were counted and expressed normalized to the media area.

### Statistical analyses

Data are presented as mean ± standard deviation (SD) or median ± interquartile range (IQR) unless otherwise stated. Statistical analyses were conducted using GraphPad Prism software (version 8.1.2). Outliers (ROUT 2%, GraphPad Prism software) were removed before analysis. Datasets were tested for equivalence of variance via the Brown-Forsythe test or F tests and for normality with Shapiro–Wilk tests. For normally distributed data, when one experimental condition was compared to one control condition, two-tailed paired t-test was used. When comparing multiple (> 2) conditions within an experiment, a one-way ANOVA with Sidak multiple comparison test was used. Non-parametric tests (Kruskal–Wallis with Dunn’s multiple comparisons in place of ANOVA and Mann–Whitney in place of t-test) were conducted for non-normally distributed data. The criterion for statistical significance was *P* < 0.05.

## Supplementary information

Supplementary Information

## Data Availability

Raw RNAseq data and gene-level counts have been uploaded onto the NCBI Gene Expression Omnibus database (GEO, GSE142417). Data relating to IL11 stimulation (no significant changing genes) has not been uploaded. The authors declare that all other data supporting the findings of this study are available within the paper and its Supplementary Information online. Any additional information is available upon reasonable request to the corresponding author.
