# Supplementary material for: Interleukin-11 is important for vascular smooth muscle phenotypic switching and aortic inflammation, fibrosis and remodeling in mouse models
Source: Sci Rep. 2020 Oct 20;10:17853. doi: 10.1038/s41598-020-74944-7 (PMC7576123; doi:10.1038/s41598-020-74944-7)
Supplement: Supplementary file 1 — Supplementary Information [file 41598_2020_74944_MOESM1_ESM.pdf]

SUPPLEMENTARY INFORMATION

Interleukin-11 is important for vascular smooth muscle phenotypic switching and aortic inflammation, fibrosis and remodeling in mouse models

Wei-Wen Lim, PhD[1,2‡], Ben Corden, MRCP, PhD[1,2,3,4‡], Benjamin Ng, PhD[1,2], Konstantinos Vanezis, PhD[3,4], Giuseppe D'Agostino, PhD[2], Anissa A. Widjaja, PhD[2], Wei-Hua Song, PhD[1], Chen Xie, BSc[1], Liping Su, BSc[1], Xiu-Yi Kwek, BSc[1], Nicole G.Z. Tee, MSc[1], Jinrui Dong, PhD[2], Nicole S.J. Ko, BSc[2], Mao Wang, BSc[2], Chee Jian Pua, BSc[1], Muhammad H. Jamal, BSc[1], Beeyong Soh, BSc[1], Sivakumar Viswanathan, PhD[2], Sebastian Schafer, PhD[1,2], Stuart A. Cook, MRCP, PhD[1,2,3,4*]

‡ These authors contributed equally to this work

SUPPLEMENTARY METHODS

Reagents

Magnetic micro-beads for cell-specific depletion includes human CD90 (130-096-253) and CD144 (130-097-857), mouse CD45 (130-052-301), CD90.2 (130-049-101) and CD31 (130-097-418) purchased from Miltenyi Biotec. The following recombinant proteins were used: TGFβ1 (mouse: 7666-MB, R&D Systems; human: PHP143B, Bio-Rad), IL11 (mouse: Z03052, GenScript; human: PHC0115, Life Technologies), ANGII (A9525, Sigma-Aldrich). U0126 (9903S, Cell Signaling) was used as a selective inhibitor of MEK/ERK signaling. Neutralizing IL11 (X203 clone) and IgG isotype control was produced by Aldevron. The following primary antibodies were used: CD90 (ab181469), myosin light chain (ab79935), CD144 (ab33168), smooth muscle myosin heavy chain 11 (SMMHC; ab53219), transgelin (SM22α; ab14106), myocardin (ab203614), αSMA (ACTA2; ab5694), IL11RA (ab125015), IL6R (ab222101), Collagen I (ab34710) from Abcam; COL1A1 (sc-293182), COL3A1 (sc-271249) from Santa Cruz; GAPDH (2118), p-ERK (4370), ERK (4695), p-STAT3 (4113) and STAT3 (4904) from Cell Signaling, IL11 (PA5-36544, Invitrogen), LGALS3 (CL8942AP, Cedarlane), LAMP2 (550292, BD Bioscience). Isotype controls included normal rabbit IgG (ab27478, Abcam) and normal rat IgG (sc-2026, Santa Cruz). Secondary antibodies include anti-rabbit Alexa Fluor-555 or anti-mouse Alexa Fluor-647 antibodies (A21429 and A21236 respectively, Invitrogen), anti-rabbit Alexa Fluor-488 (ab150077, Abcam), anti-rabbit HRP (7074, Cell Signaling) and anti-mouse HRP (7076, Cell Signaling), anti-rabbit IgG peroxidase (A0545, Sigma-Aldrich) and anti-rat IgG peroxidase (MP-7404, Vector Lab).

Human VSMCs Cell Culture

Patients aged ≥21 and ≤81 years of age undergoing coronary bypass grafting at the National Heart Centre Singapore were recruited to the study. Patients with valvular heart disease or previous atrial intervention were excluded. Aortic biopsies and/or left internal mammary artery (LIMA) trimmings were obtained and used to outgrow primary aortic and arterial VSMCs respectively. Vascular biopsies were prepared by the removal of the tunica adventitia and endothelium under a dissecting microscope. The tunica media was minced into 1-2 mm^3 pieces and explanted onto 60 mm cell culture dishes. VSMCs were maintained in complete M231 medium (M-231-500) with smooth muscle growth supplement (S-007-25) and 1% antibiotic-antimycotic (15240062) from Life Technologies, in a humidified atmosphere at 37°C and 95% air/5% CO_2. At sub-maximal confluence, cells were passaged by detachment with Accutase (A6964, Sigma-Aldrich) for subculture. At passage 1-2, VSMCs were negatively selected by magnetic separation with LD columns (130-042-901, Miltenyi Biotec) for fibroblast-expressing CD90 and endothelial cell-expressing CD144 for experiments according to manufacturer's instructions.

Experiments were carried out at low cell passages (≤ passage 4) and cells were growth restricted with 0.2% fetal bovine serum (FBS) in basal M231 for 24h before treatment with recombinant human IL11 (5 ng/ml), TGFβ1 (5 ng/ml), or ANGII (100nM) in serum-free M231 for 24h. Unstimulated VSMCs for the same duration under basal M231 medium was included as controls. For MEK/ERK inhibition studies, VSMCs were stimulated with IL11 in the presence of U0126 (10 µM) reconstituted in dimethyl sulfoxide (DMSO) compared to vehicle controls.

RNA-sequencing

Total RNA was isolated using Trizol Plus RNA mini kit (12183555, Life Technologies) and quantified using Qubit RNA high sensitivity assay kit (Life Technologies). RNA integrity number (RIN) was determined with the LabChip GX RNA Assay Reagent Kit (PerkinElmer). TruSeq Stranded mRNA Library Prep kit (Illumina) was used to assess transcript abundance following standard manufacturer’s instructions. Briefly, poly(A)+ RNA was purified from 0.8 - 1 µg of total RNA with RIN > 7, fragmented, and used for cDNA synthesis, followed by 3' adenylation, adaptor ligation, and PCR amplification. The final libraries were quantified using KAPA library quantification kits (KAPA Biosystems) on StepOnePlus Real-Time PCR system (Applied Biosystems). The quality and average fragment size of the final libraries were determined using LabChip GX DNA High Sensitivity Reagent Kit (PerkinElmer). Libraries were pooled and sequenced on a NextSeq 500 benchtop sequencer using 75-bp paired-end sequencing chemistry. Raw sequencing data (.bcl files) were demultiplexed into individual FastQ read files with Illumina’s bcl2fastq v2.16.0.10 based on unique index pairs. The adaptor sequences and low-quality reads/bases were trimmed using Trimmomatic v0.36[59] and the read quality was assessed with FastQC v0.11.5. High-quality reads were mapped to Ensembl human GRCh38 v92 reference genome using Spliced Transcripts Alignment to a Reference (STAR) v2.5.2b[60]. STAR alignment options were --outFilterType BySJout --outFilterMultimapNmax 20 --alignSJoverhangMin 8 --alignSJDBoverhangMin 1 --outFilterMismatchNmax 999 --alignIntronMin 20 --alignIntronMax 1000000 --alignMatesGapMax 1000000. Strand-specific raw counts of uniquely mapped reads (paired-end) were summarized with featureCounts to get gene-level quantification of genomic features: featureCounts -t exon -g gene_id -s 2 –p. Differential expression analyses were performed in R 3.5.2

using the Bioconductor package DESeq2 1.22.2, using the Wald test for comparisons and including the variance shrinkage step setting a significance threshold of 0.05. Genes with less than 1 count in all samples were removed to speed up the analysis. The sample ID was included in the model to account for inter-patient variability and the unstimulated condition was always used as reference.

For GO over-representation analysis, differentially expressed genes (FDR < 0.05) were tested for statistically significant over-representation against the background of all expressed genes using the R package gProfileR[61] with “strong” hierarchical filtering. Gene Set Enrichment Analyses (GSEA) were run using the fgsea library[62], pre-ranking the gene list by the “stat” column of the DESeq2 results output and using 10^5 permutations.

FANTOM5 data processing

Gene expression data in primary cell types with replicates were downloaded from the FANTOM5 web resource[22]. Since the FANTOM5 data is at the level of transcription start site expression derived from Cap Analysis of Gene Expression (CAGE) sequencing, we calculated the gene level expression by summing all counts that were assigned to a given gene. These were normalized by library size to calculate the tags per million (TPM) for each gene. To compare the expression profiles of IL11RA and IL6R, we extracted the TPM for these two genes across 511 different primary cell types encompassing cell types from all lineages. In each case, for which the expression of either IL11RA or IL6R is above the level of noise, we highlight these cell types and categorize them as described in the FANTOM5 cell type ontology.

Immunofluorescence and confocal microscopy

VSMCs cultured in 8-well ibiTreat μ-slides (Ibidi) were fixed with 4% paraformaldehyde (PFA), permeabilized with 0.1% Triton X-100 and blocked with 1% bovine serum albumin (BSA) before incubation with primary antibodies for CD90 (1:200), myosin light chain (1:150), CD144 (1:150), SMMHC (1:150), and SM22α (1:200) at 4°C overnight. Cells were visualized using either anti-rabbit Alexa Fluor-555 or anti-mouse Alexa Fluor-647 antibodies (both 1:1000). Samples were stained with Alexa Fluor-488 phalloidin (1:200; A12379, Invitrogen) to visualize actin filaments. For immunofluorescence staining for IL11RA and IL6R, VSMCs were incubated with either anti-IL11RA (1:200), anti-IL6R (1:200) or without primary antibody at 4 °C overnight and visualized with anti-rabbit Alexa Fluor-488 (1:200). Cell nuclei were stained with DAPI (1:1000; D1306, Invitrogen) and cells mounted with VECTASHIELD mounting medium (Vector Laboratories). Images were acquired using a confocal laser scanning microscope (LSM 710, Zeiss).

Flow cytometry

Human aortic and arterial VSMCs (Passage 3) were prepared as before. VSMCs were dissociated and fixed in 4% PFA, PBS washed, and non-specific sites blocked with 5% BSA. Cells were incubated with either anti-IL11RA (1:200) or anti-IL6R (1:200) for 1h, PBS washed, and probed with anti-rabbit Alexa Fluor-488 (1:200) for 1h. Cells were quantified using flow cytometry (Fortessa, BD Biosciences) and analyzed with FlowJo version 10 software (Tree Star).

Operetta high content immunofluorescence analysis

High-content immunofluorescence imaging and quantification of VSMC activation were performed as previously described[14]. VSMCs were seeded in 96-well CellCarrier plates (6005550, PerkinElmer) at a density of $5x10^3$ cells per well and following experimental conditions, fixed and permeabilized as before. To measure cell proliferation, 5-Ethynyl-2´-deoxyuridine (EdU; 10 µM) was added 1h post-stimulation and detected after 24h of stimulation using the Click-iT EdU Labelling kit (C10350, Life Technologies) according to manufacturer's protocol. Cells were blocked using 0.5% BSA and 0.1% Tween-20 in PBS before incubation with primary anti-Collagen I (1:500), anti-SM22α (1:200), and anti-myocardin (1:200) and visualized using Alexa Fluor-488-conjugated secondary antibody (1:1000). Cells were counterstained with DAPI (D1306, Invitrogen) in blocking solution. Plates were scanned and images were collected with the Operetta high-content imaging system 1483 (PerkinElmer). The measurement of collagen I fluorescence intensity per area was done using Columbus software version 2.7.1 (PerkinElmer). Fluorescence intensity (collagen I and myocardin expression) was normalized to the number of cells detected in the field and recorded for seven fields per well. EdU^{+ve} and $SM22α^{+ve}$ cells were quantified using Harmony software version 3.5.2 (PerkinElmer) and the percentage of VSMCs was determined for each field.

Scratch wound migration assay

Scratch wound assays were performed on fully confluent monolayers of human and murine VSMCs (passage 3-4) cultured on 35mm culture dishes. After synchronizing in low serum media (0.2% FBS-M231) for 24h, a linear scratch was created with a sterile pipette tip followed by treatment with: either IL11 (5 ng/ml), TGFβ1 (5 ng/ml) or ANGII (100 nM) with or without either IL11 neutralizing antibody (2 µg/ml, X203;

Aldevron) or IgG isotype control (2 µg/ml, IgG; Aldevron) for 24h (human VSMCs) to 48h (murine VSMCs). The wound area was imaged at 0h and 24h or 48h and quantified using ImageJ software with the MRI wound healing tool plugin (http://dev.mri.cnrs.fr/projects/imagej-macros/wiki/Wound_Healing_Tool). Migration of VSMCs was calculated as a percentage of wound area recovered at endpoint compared to 0h. A total of 6 to 10 random regions were analyzed per treatment and averages reported.

Invasion assay

The invasive capacity of VSMCs were assessed using Boyden chamber invasion assays (Cell Biolabs). VSMCs were cultured in serum-free M231 medium for 24h prior to cell invasion assays. Equal numbers of VSMCs were seeded in duplicates onto apical chambers containing Matrigel for invasion assays. VSMCs invaded towards the bottom chamber containing 2% FBS-M231 medium with the respective chemoattractant. After 24h of incubation, media was removed, and non-invasive cells were removed using cotton swabs. Invaded cells were stained with cell staining solution (Cell Biolabs) and colorimetrically quantified at 540 nm. For antibody inhibition studies, VSMCs were pre-treated with X203 or IgG control antibodies (both 2 µg/ml) for 15 min prior to addition of chemoattractant.

Mouse models

IL11 receptor null primary VSMCs

Four-to-six week old mice lacking functional alleles for *Il11ra1* (*Il11ra1*$^{-/-}$, KO) and wild-type littermates (*Il11ra1*$^{+/+}$, wildtype) were used for aortic VSMC extraction and culture using a modified protocol adapted from published literature[63,64]. The thoracic

aorta were minced, digested for 45 minutes in M231 medium containing 1% antibiotic-antimycotic and 0.25 mg/ml Liberase TM (Roche) with mild agitation at 37°C and subsequently explant-cultured in complete M231 medium. Mixed cells were outgrown from digested aortic tissue and at 80-90% confluence at passage 2, VSMCs were enriched via negative selection with micro-beads for CD45 (leukocytes), CD90.2 (fibroblasts), and CD31 (endothelial cells;) using the MidiMACS separator (130-042-302, Miltenyi Biotec).

Passage 3 to 4 mouse aortic VSMCs were used for scratch wound migration assay and cultured for collagen secretion assay. Murine VSMCs were either treated with recombinant mouse TGFβ1 (5 ng/ml), IL11 (5 ng/ml), or ANGII (100nM) in M231 basal medium.

Smooth-muscle specific IL11 overexpressing mice

Heterozygous *Rosa26-IL11* (C57BL/6N-*Gt(ROSA)26Sor*$^{tm1(CAG\text{-}Il11)Cook}$/J) mice[14] were crossed to the hemizygous *Myh11-CreERT2* (B6.FVB-Tg(Myh11-cre/ERT2)1Soff/J) mice[29] (Jackson Laboratory; 031928 and 019079 respectively) to generate double heterozygous *Myh11*^{CreERT2}*:Rosa26*$^{IL11/+}$ offspring. *Myh11*^{CreERT2}*:Rosa26*$^{IL11/+}$ mice (*n* = 33) were injected with 3 doses of 50 mg/kg tamoxifen (TAM; T5648, Sigma-Aldrich) IP at 6 weeks of age to induce Cre-mediated *Il11* transgene induction (referred to as *Il11*-Tg mice). 20 mice died or were sacrificed prior to endpoint (14 days post-TAM) due to inflammatory bowel disease[30], resulting in 13 survivors. Control littermates were injected with an equal amount of corn oil (C8267, Sigma-Aldrich) vehicle (*n* = 12). Mice were euthanized at 14 days from the first TAM dose.

Transverse aortic constriction (TAC)

TAC surgeries were performed on C57BL/6J male mice (*n* = 24) purchased from *In Vivos Pte Ltd*, Singapore as described[65]. Age-matched sham controls underwent the same operative procedure without ligation (*n* = 10). Trans-thoracic two-dimensional Doppler echocardiography confirmed increased pressure gradients (>40 mmHg) indicative of successful TAC. Mice were randomized to receive post-operative antibody treatment conducted by IP injections of either X203 or IgG control antibodies (*n* = 12 per group) at a dose of 20 mg/kg twice per week for two consecutive weeks starting 24h following TAC. Mice were euthanized at 2 weeks post-TAC prior to maladaptive heart failure response[65] and the proximal ascending aorta was excised.

Angiotensin II (ANGII) infusion

ANGII pump infusions were performed as previously described[14]. C57BL/6J male mice were implanted SQ with an osmotic minipump (Alzet model 1004, Durect) containing either angiotensin II (ANGII, 2 mg/kg per day; *n* = 28) or an identical volume of saline for controls. ANGII-treated mice were randomized to receive post-operatively injections of either X203 or IgG control antibodies at a dose of 20 mg/kg twice per week for four consecutive weeks. Mice underwent aortic echocardiography and were euthanized at 4 weeks post-ANGII and the thoracic aorta was excised. No mortality associated with ANGII infusion was observed.

Aortic Echocardiography

In vivo trans-thoracic echocardiography was conducted using Vevo 2100 with a MS400 linear array transducer (VisualSonics), 18-38 MHz by a trained

echocardiographer (NGZT) blinded to genotype and treatment groups. Mice were anaesthetized with 3% isoflurane and maintained at 1% isoflurane with the body temperature maintained at 37°C on a heated platform. Chest and neck hair were removed using depilatory cream and a layer of acoustic coupling gel was applied to the thorax. Aortic root and ascending aortic diameters were assessed from *B* and *m*-mode of parasternal long-axis view, using inner edge-to-inner edge according to USA and European guidelines[66]. Peak aortic flow velocity was obtained by applying pulsed-wave Doppler across the aortic valve from the aortic arch at suprasternal view. All measurements were averaged over three cardiac cycles. Aortic dimensions were referenced to body weight per animal to account for differences in body mass.

Enzyme-linked immunosorbent assay

VSMCs were seeded at 10,000 live cells per cm^2 in 35mm culture dishes and maintained on complete M231 medium as previously mentioned. Following 0.2% FBS-M231 growth restriction, VSMCs were stimulated with 1 ml of TGFβ (5 ng/ml) or ANGII (100nM) as previously described. The levels of IL11 in equal volumes of VSMC culture medium were quantified with the human IL11 Quantikine ELISA kit (D1100, R&D Systems) and normalized against unstimulated conditions. The levels of TIMP-1 and MMP2 in cell supernatant were quantified using the human TIMP-1 and MMP2 Quantikine ELISA kit (DTM100 and MMP200, R&D Systems) respectively. All assays were conducted as per manufacturer's instructions.

Colorimetric Assays

To detect for secreted collagen by VSMCs, cell culture supernatant was first concentrated using polyethylene glycol solution (90626, Chondrex) prior to

quantification using the Sirius red collagen detection kit (9062, Chondrex) according to manufacturer's instructions.

Immunoblotting

Western blot analysis was carried out on total protein extracts from human VSMCs or mouse thoracic aorta stripped of periaortic fat and adventitia under a dissecting microscope. Cells were lysed with RIPA lysis buffer (containing protease and phosphatase inhibitors (Roche)) on ice. Cell scrapers were used to scrape the cells and lysate extracted. Snap-frozen aortic tissues were homogenized by gentle rocking in RIPA lysis buffer followed by centrifugation to clear the lysate. Protein concentrations were determined by bicinchoninic acid method (23225, Pierce). Equal amounts of protein lysates were separated by SDS–PAGE, transferred to a polyvinylidene difluoride (PVDF) membrane, and incubated overnight with primary antibodies for IL11 (1:5000; X203), ACTA2 (1:1000), COL3A1 (1:1000), GAPDH (1:5000), p-ERK1/2 (1:1000), ERK1/2 (1:1000), p-STAT3 (1:1000) and STAT3 (1:1000). Protein bands were visualized on the ChemiDoc MP imaging system (Bio-Rad) with automated exposure settings for intense bands using the ECL detection system (Pierce) with the appropriate secondary antibodies: anti-rabbit HRP (1:1000) or anti-mouse HRP (1:1000). Uncropped blots are demonstrated in Supplementary Fig. S12 to S14 online.

Quantitative polymerase chain reaction (qPCR)

Total RNA was extracted from snap-frozen tissues using RNAzol RT (Sigma-Aldrich) followed by Purelink RNA mini kit (Invitrogen) purification. The cDNA was synthesized using iScript cDNA synthesis kit (Bio-Rad) as per manufacturer's

instructions. Gene expression analysis was performed in duplicates using fast SYBR green (Qiagen) technology on the ViiA 7 real-time PCR system (Applied Biosystems). Expression data were normalized to *Gapdh* mRNA expression levels and fold-change was calculated using the $2^{-\Delta\Delta Ct}$ method. The primer sequences are available in Supplementary Table S3 online.

Histology

Transverse sections (5 µm) of paraffin-embedded proximal ascending aorta were used for histological stains. Sections were stained with Masson's Trichrome (HT15, Sigma-Aldrich) for collagen, hematoxylin and eosin (H&E) for nuclei, and Verhoeff Van Gieson (VVG; 87017, Thermo Fisher) for elastin. Brightfield photomicrographs were randomly captured by a researcher (XYK) blinded to the treatment groups using the Olympus BX51 microscope and Image-Pro Premier 9.2 (Media Cybernetics).

Total fibrosis was measured by ImageJ (v1.52a, NIH) with *Color deconvolution*-Masson Trichrome vector (programmed by Landini) in photomicrographs taken at 100X magnification of each section (*n* = 5-6 per group) and expressed as a percentage of collagen stained area over total tissue area. Media thickness was measured as the intima-media distance using the incremental distance tool at a calibrated step size of 10µm on Image-Pro Premier 9.2 (Media Cybernetics) and reported as an average of 62-142 measurements across 4 random photomicrographs (400X magnification) per section (*n* = 5-6 per group).

Adventitial area and smooth muscle cell (SMC) nuclei were quantified in H&E stained sections as described[67] across 4 random photomicrographs (400X magnification) per section (*n* = 5-6 per group). *Color deconvolution*-H&E vector was performed. The adventitial area was expressed as a percentage of total tissue area. SMC nuclei were counted with the *Cell Counter* plugin (programmed by De Vos) and expressed as nuclei counts over media area. Elastin area and elastin breaks were quantified in VVG stained sections with *color deconvolution*-ROI vector selecting for elastin across 4 random photomicrographs (400X magnification) per section (*n* = 5-6 per group). Elastin area fraction was expressed as the elastin positive area over total tissue area. Elastin breaks were counted and expressed normalized to the media area.

Transverse aortic sections were deparaffinized, permeabilized with Triton X-100 (Sigma-Aldrich) and antigen retrieved with Bull's Eye Decloaker (Biocare Medical) for immunohistochemistry. Slides were then blocked for endogenous peroxidase with Bloxall blocking solution (Vector Lab) followed by blocking with either 3% BSA, or mouse on mouse blocking reagent (Vector Lab). Anti-IL11 (1:100), ACTA2 (1:500), SM22α (1:100), LGALS3 (1 μg/ml) and LAMP2 (1 μg/ml) were added and incubated overnight at 4°C. Anti-rabbit (1:100) and anti-rat IgG (1 μg/ml) isotype controls were added as respective negative controls (see Supplementary Fig. S11). Slides were incubated with anti-rabbit IgG peroxidase (1:500) and anti-rat IgG peroxidase (MP-7404) followed by chromogen development with ImmPACT DAB peroxidase substrate kit (SK-4105, Vector Lab) according to manufacturer's instructions. Lastly, Gill's hematoxylin (H-3401, Vector Lab) was added for nuclear counterstain.

Statistical Analyses

Data are presented as mean ± standard deviation (SD) or median ± IQR unless otherwise stated. Statistical analyses were conducted using GraphPad Prism software (version 8.1.2). Outliers (ROUT 2%, GraphPad Prism software) were removed before analysis. Datasets were tested for equivalence of variance via the Brown-Forsythe test or F tests and for normality with Shapiro-Wilk tests. For normally distributed data, when one experimental condition was compared to one control condition, two-tailed paired t-test was used. When comparing multiple (> 2) conditions within an experiment, a one-way ANOVA with Sidak multiple comparison test was used. Non-parametric tests (Kruskal-Wallis with Dunn’s multiple comparisons in place of ANOVA and Mann-Whitney in place of t-test) were conducted for non-normally distributed data. The criterion for statistical significance was $P < 0.05$.

SUPPLEMENTARY TABLES

Supplementary Table S1. Patient undergoing coronary artery bypass grafting who consented to donate aortic and/or left internal mammary artery biopsies for explant-culture VSMCs

	Aortic (n = 10)	LIMA (n = 22)
**DEMOGRAPHICS**		
Age (Years)	55.7 ± 9.5	56.4 ± 8.6
Race (CH / MY / IN / OTHERS)	4 / 5 / 0 / 1	11 / 8 / 1 / 2
Sex (M / F)	8 / 2	20 / 2
Height (m)	1.6 ± 0.08	1.7 ± 0.1
Weight (kg)	74.8 ± 23.6	71.4 ± 16.1
Systolic BP (mmHg)	130.5 ± 23.5	126.6 ± 20.9
Diastolic BP (mmHg)	67.9 ± 8.19	68.8 ± 10.4
**MEDICAL HISTORY**		
Hypertension (Y / N / Unk)	6 / 4	14 / 6 / 2
Smoking (Y / N / Ex)	3 / 4 / 3	4 / 7 / 11
Myocardial infarct (Y / N / Unk)	0 / 10 / 0	1 / 19 / 2
*Atrial fibrillation (Y / N)	0 / 10	1 / 21
Diabetes (Y / N)	3 / 7	14 / 8
Diuretic (Y / N / Unk)	0 / 10	1 / 20 / 1
Beta Blocker (Y / N)	10 / 0	22 / 0
Nitrate (Y / N)	7 / 3	15 / 7
Insulin (Y / N)	0 / 10	1 / 21
ACE or ARB (Y / N)	2 / 8	4 / 18
Statin (Y / N)	9 / 1	20 / 2
Calcium antagonist (Y / N)	0 / 10	1 / 21
**BLOOD TESTS**		
Hemoglobin (g/L)	13.2 ± 2.0	13.6 ± 1.5
Urea (mM/L)	5.61 ± 3.8	5.59 ± 2.67
Creatinine (µM/L)	95.1 ± 43.8	91.5 ± 32.4

LIMA refers to the left internal mammary artery. Categorical data are presented as yes (Y) or no (N), unless otherwise indicated. *Atrial fibrillation refers to any present or previous history of atrial fibrillation. Quantitative data represented as mean ± SD. CH, Chinese; MY, Malay; IN, Indian; Ex, ex-smoker; Unk, unknown.

Supplementary Table S2. Top 20 genes up- and down-regulated with TGFβ1 stimulation in aortic and arterial VSMCs

	**Aortic VSMC**						**Arterial VSMC**					
	**Upregulated**			**Downregulated**			**Upregulated**			**Downregulated**		
No	**Gene**	**log_2 (FC)**	**adj. *P*-value**	**Gene**	**log_2 (FC)**	**adj. *P*-value**	**Gene**	**log_2 (FC)**	**adj. *P*-value**	**Gene**	**log_2 (FC)**	**adj. *P*-value**
1	ST6GAL2	3.15	2.38E-33	GDF5	-2.21	5.54E-29	CILP	4.49	4.80E-40	PKD1L2	-3.11	1.20E-20
2	LEFTY2	2.87	1.65E-22	KCNS2	-1.83	1.81E-11	LEFTY2	3.72	7.30E-32	EVI2B	-2.90	6.21E-23
3	LDLRAD4	2.81	6.14E-37	SLC2A12	-1.80	2.59E-26	KANK4	3.63	5.76E-19	OMG	-2.89	6.80E-16
4	TSPAN2	2.67	3.35E-21	ITGB8	-1.73	9.41E-19	ACTC1	3.55	3.57E-20	FAM65C	-2.85	1.79E-13
5	**IL11**	**2.66**	**1.65E-22**	LGR5	-1.70	1.90E-12	TSPAN2	3.48	2.19E-38	COLEC12	-2.76	2.29E-19
6	RASL11B	2.65	6.14E-37	TNFRSF1B	-1.66	2.53E-09	LDLRAD4	3.33	3.96E-28	MUSK	-2.72	5.32E-15
7	COMP	2.54	1.00E-26	SLC6A4	-1.62	9.86E-08	RASL11B	3.10	7.32E-21	SLC6A4	-2.71	3.64E-18
8	CLEC18A	2.50	1.29E-26	RGS7BP	-1.60	1.21E-10	MAMDC2	3.05	8.70E-47	ADH1B	-2.71	1.19E-21
9	MYOM3	2.31	4.87E-21	SEMA3D	-1.58	1.08E-18	**IL11**	**3.04**	**1.52E-24**	TNFRSF1B	-2.70	2.40E-20
10	ISLR2	2.28	1.03E-13	LINC01085	-1.57	3.01E-07	ACTBL2	3.04	3.13E-26	SECTM1	-2.60	4.90E-25
11	KANK4	2.24	6.68E-12	GBP2	-1.51	8.96E-14	IGF1	2.87	2.92E-23	CTD-2370N5.3	-2.36	7.52E-20
12	WNT11	2.22	1.91E-13	SEMA5B	-1.47	2.09E-05	LPAR5	2.87	5.73E-13	RP11-366L20.2	-2.36	5.32E-23
13	RASL12	2.12	4.29E-11	KB-1517D11.4	-1.42	3.56E-05	MDFI	2.82	2.46E-14	GDF5	-2.32	1.19E-21
14	PMEPA1	2.06	6.41E-61	PLEKHG4	-1.40	5.22E-12	KCNH1	2.81	6.60E-18	DCLK1	-2.21	2.13E-18
15	ADAMTS4	2.04	2.55E-24	METTL7A	-1.39	4.90E-07	AMZ1	2.76	1.37E-15	SEC14L5	-2.21	1.02E-12
16	KRT16	2.03	1.98E-12	RXFP1	-1.38	1.84E-06	COMP	2.67	1.59E-25	EVI2A	-2.16	7.15E-16
17	HEYL	2.02	2.31E-13	SECTM1	-1.38	6.51E-07	RP11-1260E13.4	2.67	5.16E-12	CLDN11	-2.15	4.35E-19
18	MYOZ1	2.02	1.36E-11	EVI2A	-1.38	1.46E-05	ST6GAL2	2.66	2.03E-15	CYP4X1	-2.14	3.89E-16
19	CLEC18B	1.97	3.35E-15	MYOZ2	-1.38	3.94E-07	CNN1	2.65	4.16E-28	RP11-89N17.4	-2.14	7.00E-06
20	PPP1R14C	1.94	4.43E-11	SLC40A1	-1.37	1.14E-09	CLDN14	2.62	3.46E-25	MYOZ2	-2.13	1.69E-18

Supplementary Table S3. RT-qPCR primers

Gene	Forward primer (5' to 3')	Reverse primer (5' to 3')	Accession number
Human			
COL1A1	GTGCGATGACGTGATCTGTGA	CGGTGGTTTCTTGGTCGGT	NM_000088.3
COL3A1	GGAGCTGGCTACTTCTCGC	GGGAACATCCTCCTTCAACAG	NM_000090.4
ACTA2	GTGTTGCCCCTGAAGAGCAT	GCTGGGACATTGAAAGTCTCA	NM_001141945.2
CNN1	CTGTCAGCCGAGGTTAAGAAC	GAGGCCGTCCATGAAGTTGTT	NM_001299.6
MYH11	GGTCACGGTTGGGAAAGATGA	GGGCAGGTGTTTATAGGGGTT	NM_001040113.2
MYOCD	ACGGATGCTTTTGCCTTTGAA	AACCTGTCGAAGGGGTATCTG	NM_001146312.3
TAGLN	AGTGCAGTCCAAAATCGAGAAG	CTTGCTCAGAATCACGCCAT	NM_001001522.2
GAPDH	CGACAGTCAGCCGCATCTTCTTT	CCAAATCCGTTGACTCCGACCTT	NM_002046.7
Mouse			
Il11	AATTCCCAGCTGACGGAGATCACA	TCTACTCGAAGCCTTGTCAGCACA	NM_008350.4
Col1a1	GGGGCAAGACAGTCATCGAA	GTCCGAATTCCTGGTCTGGG	NM_007742.4
Col1a2	CCCAGAGTGGAACAGCGATT	ATGAGTTCTTCGCTGGGGTG	NM_007743.3
Col3a1	ATGCCCACAGCCTTCTACAC	ACCAGTTGGACATGATTCACAG	NM_009930.2
Fn1	CACCCGTGAAGAATGAAGA	GGCAGGAGATTTGTTAGGA	NM_010233.2
Timp1	GGGCTAAATTCATGGGTTCC	CTGGGACTTGTGGGCATATC	NM_001044384.1
Mmp2	ACAAGTGGTCCGCGTAAAGT	AAACAAGGCTTCATGGGGGC	NM_008610.3
Il6	AGGATACCACTCCCAACAGACC	AGTGCATCATCGTTGTTCATACA	NM_031168.2
Tnfa	CATCTTCTCAAAATTCGAGTGACAA	TGGGAGTAGACAAGGTACAACCC	NM_013693.3
Ccl2	GAAGGAATGGGTCCAGACAT	ACGGGTCAACTTCACATTCA	NM_011333.3
Ccl5	GCTGCTTTGCCTACCTCTCC	TCGAGTGACAAACACGACTGC	NM_013653.3
Acta2	TGGAGAAGCCCAGCCAGTCG	CCAGCGAAGCCGGCCTTACA	NM_007392.3
Cnn1	GCACATTTTAACCGAGGTCC	TGACCTTCTTCACAGAACCC	NM_009922.4
Myh11	TGGACACCATGTCAGGGAAA	ATGGACACAAGTGCTAAGCAGTCT	NM_001161775.1
Myocd	GATGGGCTCTCTCCAGATCAG	GGCTGCATCATTCTTGTCACTT	NM_145136.4
Tagln	TGGGCTTCCAGGTGTGGCTGAA	TTTGGTCACAGCCAAACTGCCC	NM_011526.5
Gapdh	CTGGAAAGCTGTGGCGTGAT	GACGGACACATTGGGGGTAG	NM_008084.3

SUPPLEMENTARY FIGURES

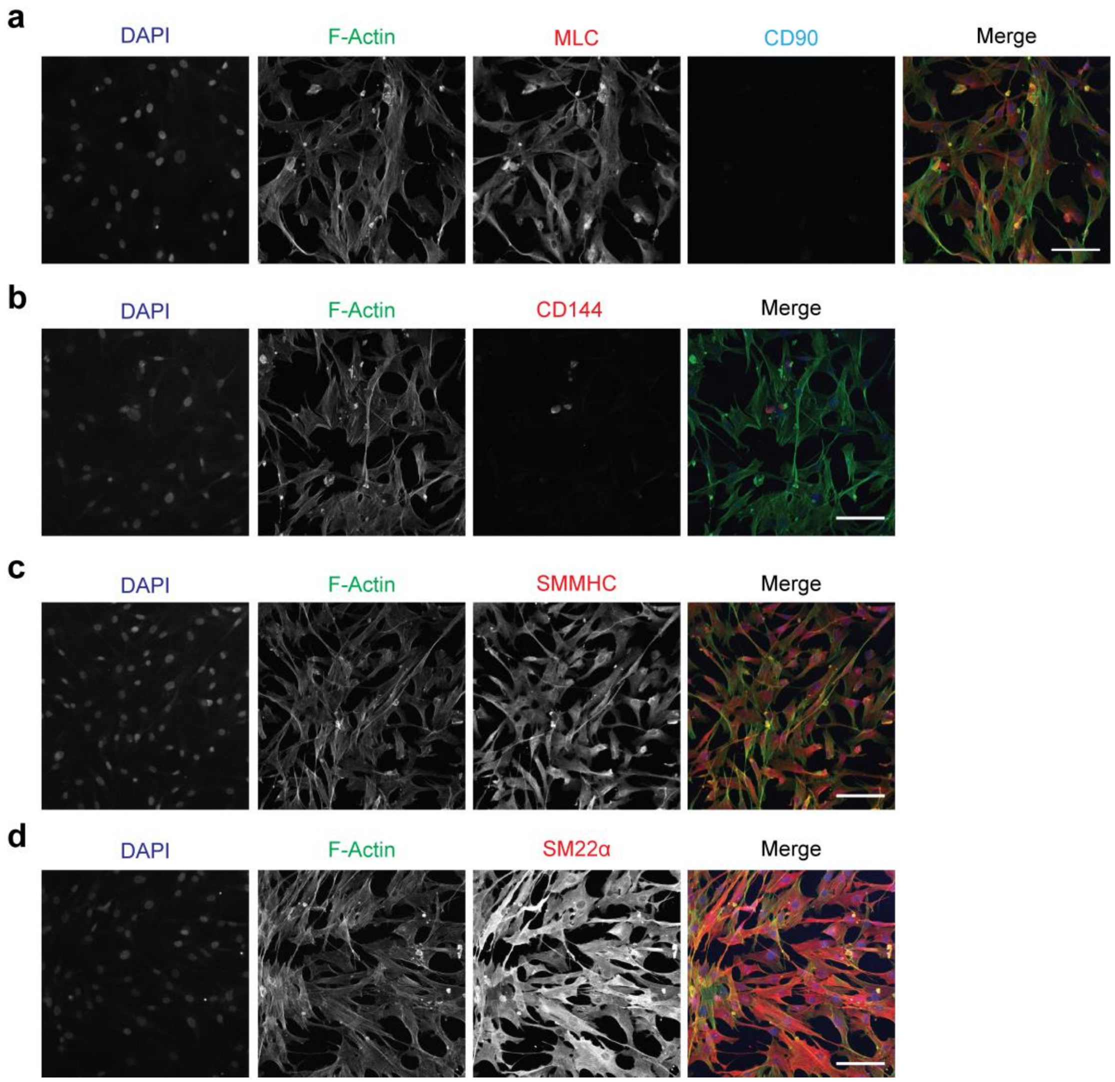

Supplementary Fig. S1. Confocal immunofluorescence of explant-cultured magnetic cell sorted human arterial VSMC (passage 3) at baseline. (**a**) VSMCs were immunostained for F-actin, myosin light chain, and Cluster of Differentiation 90 (CD90) with 4′,6-diamidino-2-phenylindole (DAPI) nuclear stain. (**b**) VSMCs were immunostained for F-actin and CD144 (endothelial cell marker) with DAPI nuclear stain. (**c**) VSMCs were immunostained for F-actin and smooth muscle myosin heavy chain (SMMHC) with DAPI nuclear stain. (**d**) VSMCs were immunostained for F-actin and transgelin (SM22α) with DAPI nuclear stain. Colored heading represents the respective pseudo-colored display in the composite images. Scale bar represents 100 µm.

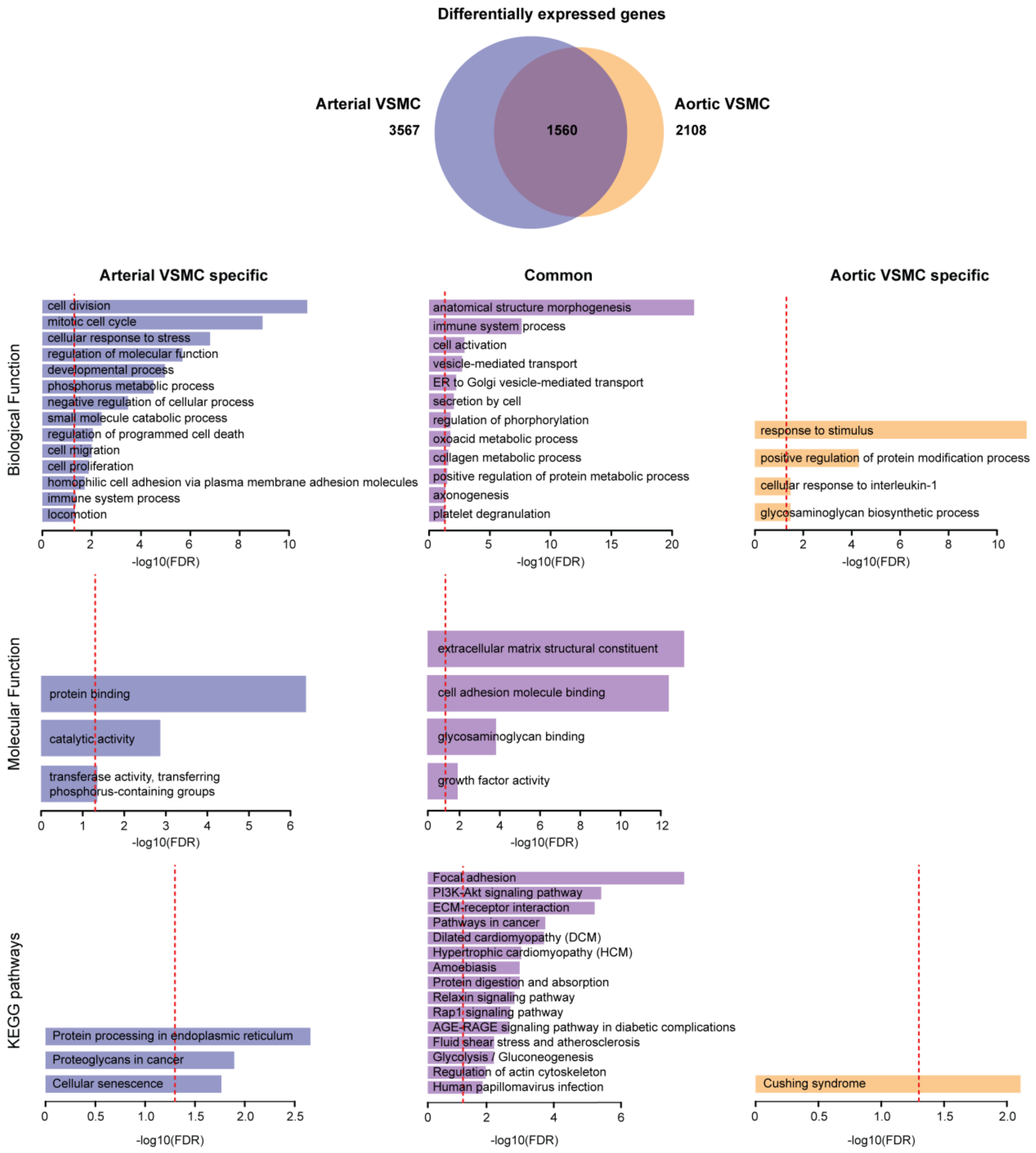

Supplementary Fig. S2. Gene ontology (GO) enrichment comparison of aortic and arterial VSMCs reveal common gene sets associated with protein production-transport. GO enrichment comparison of RNAseq data from VSMCs stimulated with TGFβ1 (5 ng/ml) for 24h in 5 biological replicates paired for aortic and arterial VSMCs. Red dotted lines indicate FDR = 0.05.

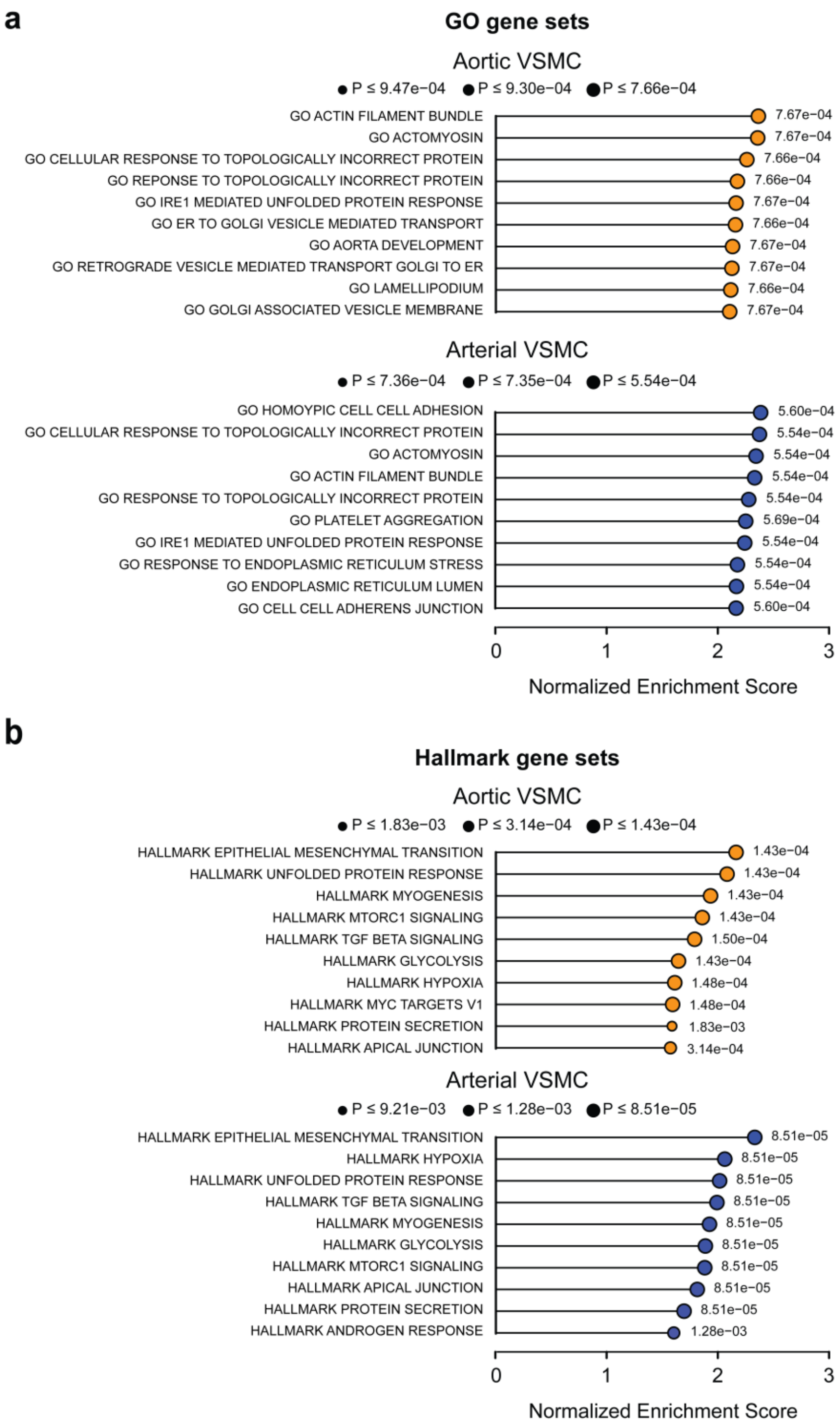

Supplementary Fig. S3. Top 10 upregulated gene set enrichment analysis (GSEA) of RNA-seq data for TGFβ1 associated transcriptional response in aortic and arterial VSMCs. (**a**) Lollipop chart of top 10 upregulated GSEA for TGFβ1 stimulated aortic and arterial VSMCs based on gene ontology (GO) gene sets. (**b**) Lollipop chart of top 10 upregulated GSEA for TGFβ1 stimulated aortic and arterial VSMCs based on MSigDB hallmark gene sets. Aortic and arterial VSMCs were obtained from the

same 5 biological replicates. Normalized enrichment scores (NES) are represented by dot sizes indicating the level of significance of FDR-adjusted *P* values.

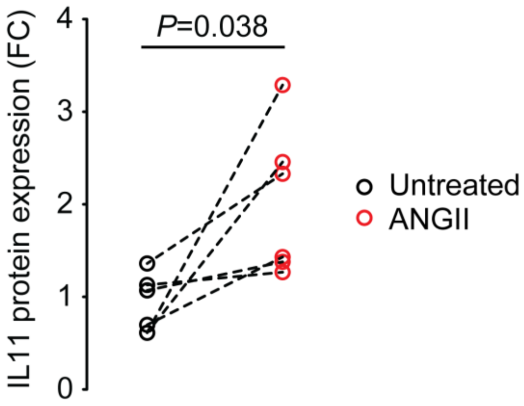

Supplementary Fig. S4. Arterial VSMCs secrete IL11 in response to ANGII stimulation. Changes in secreted IL11 levels in independent VSMC lines stimulated with and without ANGII (100 nM) for 24h in 5 biological replicates. Statistical analyses by paired t-test.

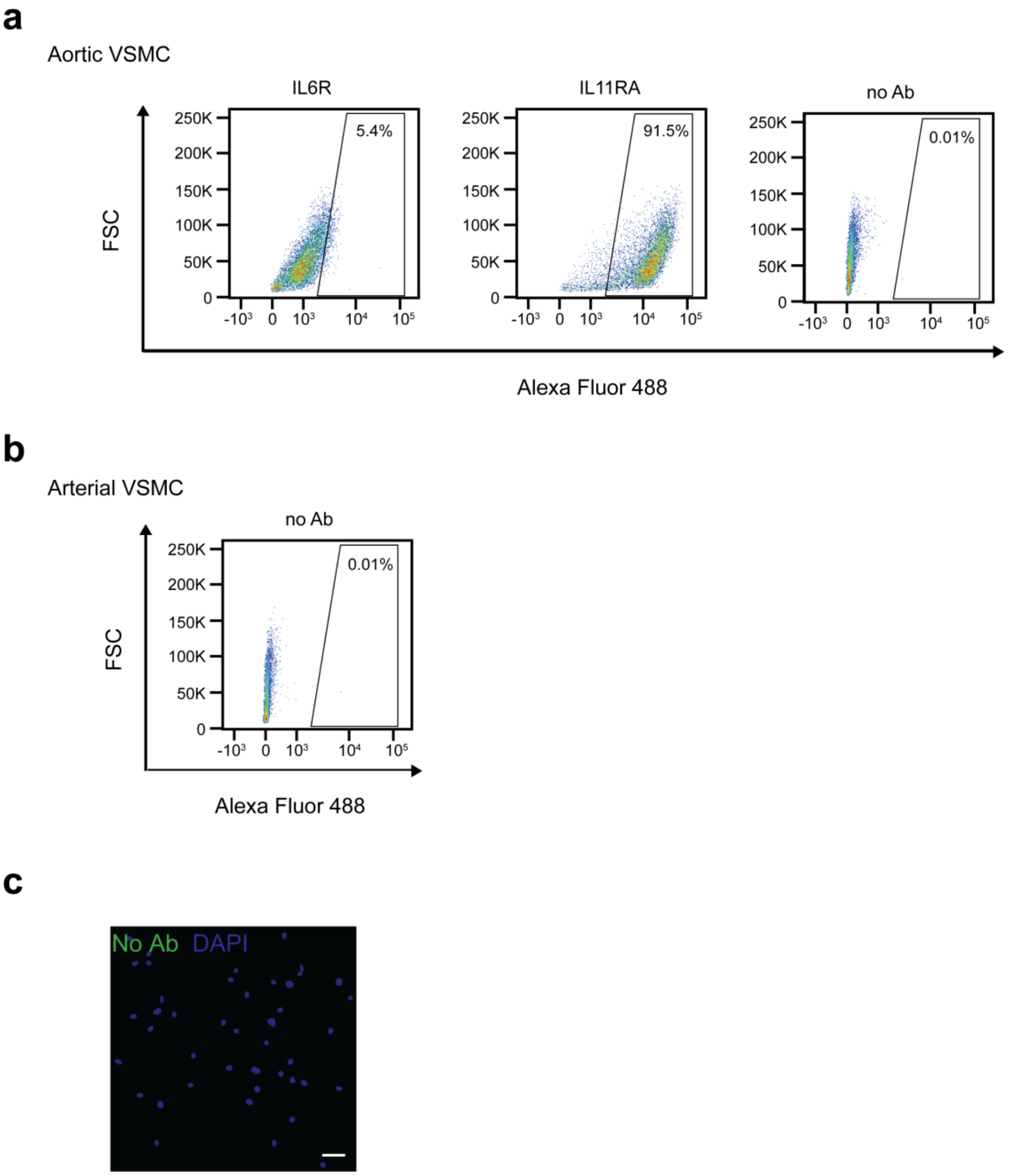

Supplementary Fig. S5. Flow cytometry of human aortic VSMCs for IL6R and IL11RA expression and no primary antibody controls for flow cytometry and immunocytochemistry of human VSMCs. (**a**) Forward scatter plots of baseline aortic VSMCs demonstrated IL11RA to be highly expressed in VSMCs and scant expression of IL6R. Absence of primary antibody (no Ab) was included as a negative control. (**b**) Absence of primary antibody was included as a negative control for images presented in Fig. 1e. (**c**) Representative immunofluorescence staining of VSMCs without primary antibody was included as a negative control for images presented in Fig. 1f. Scale bar represents 100 µm.

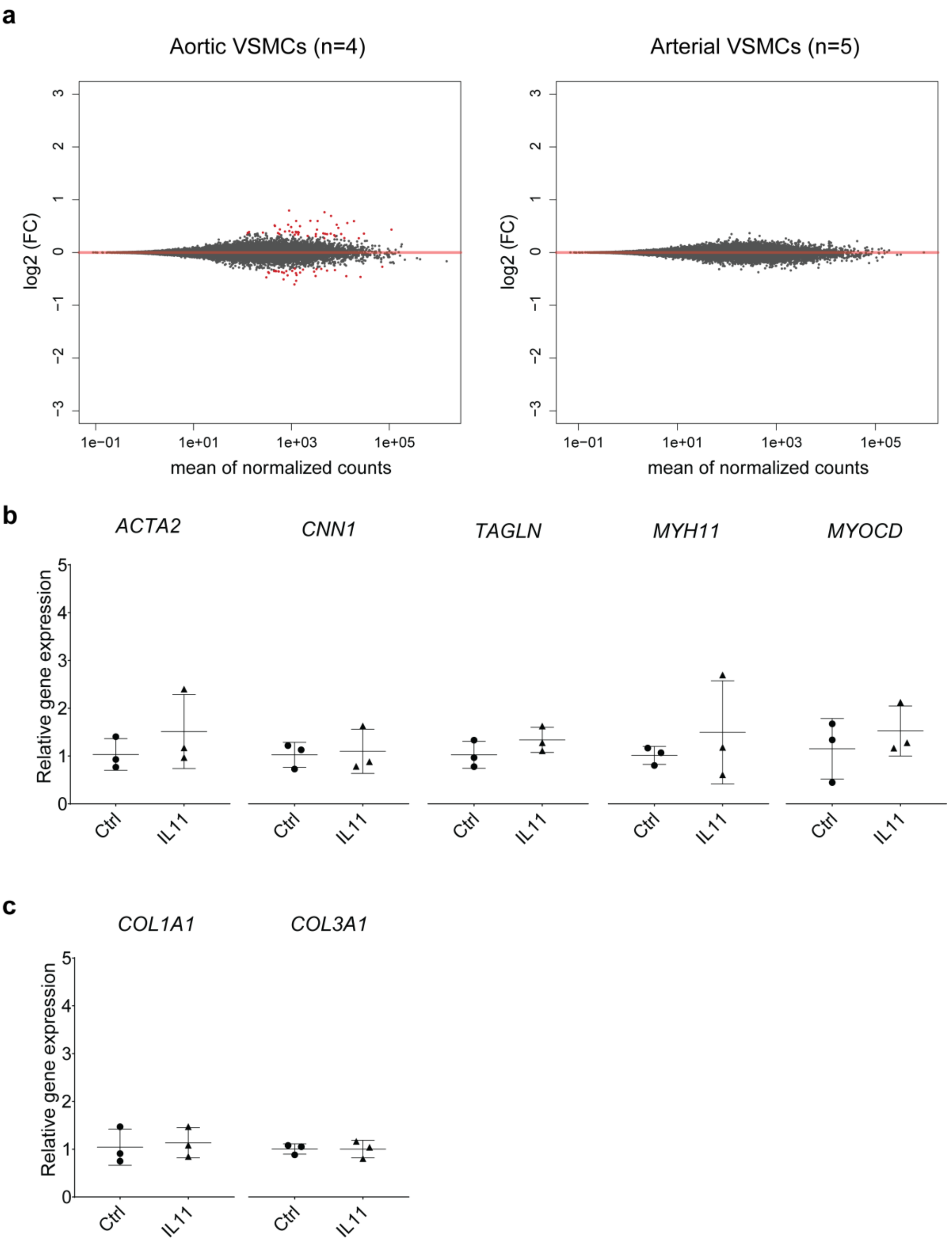

Supplementary Fig. S6. IL11 does not activate transcriptomic changes in human VSMCs *in vitro*. (**a**) RNAseq MA plots of aortic (left; *n*=4) and arterial (right; *n*=5) VSMCs were stimulated with recombinant human IL11 (5 ng/ml, 24h). Mapped read counts were normalized to unstimulated conditions in paired samples. RT-qPCR for (**b**) contractile genes and (**c**) collagen genes in human VSMCs stimulated with recombinant human IL11 (5 ng/ml, 24h).

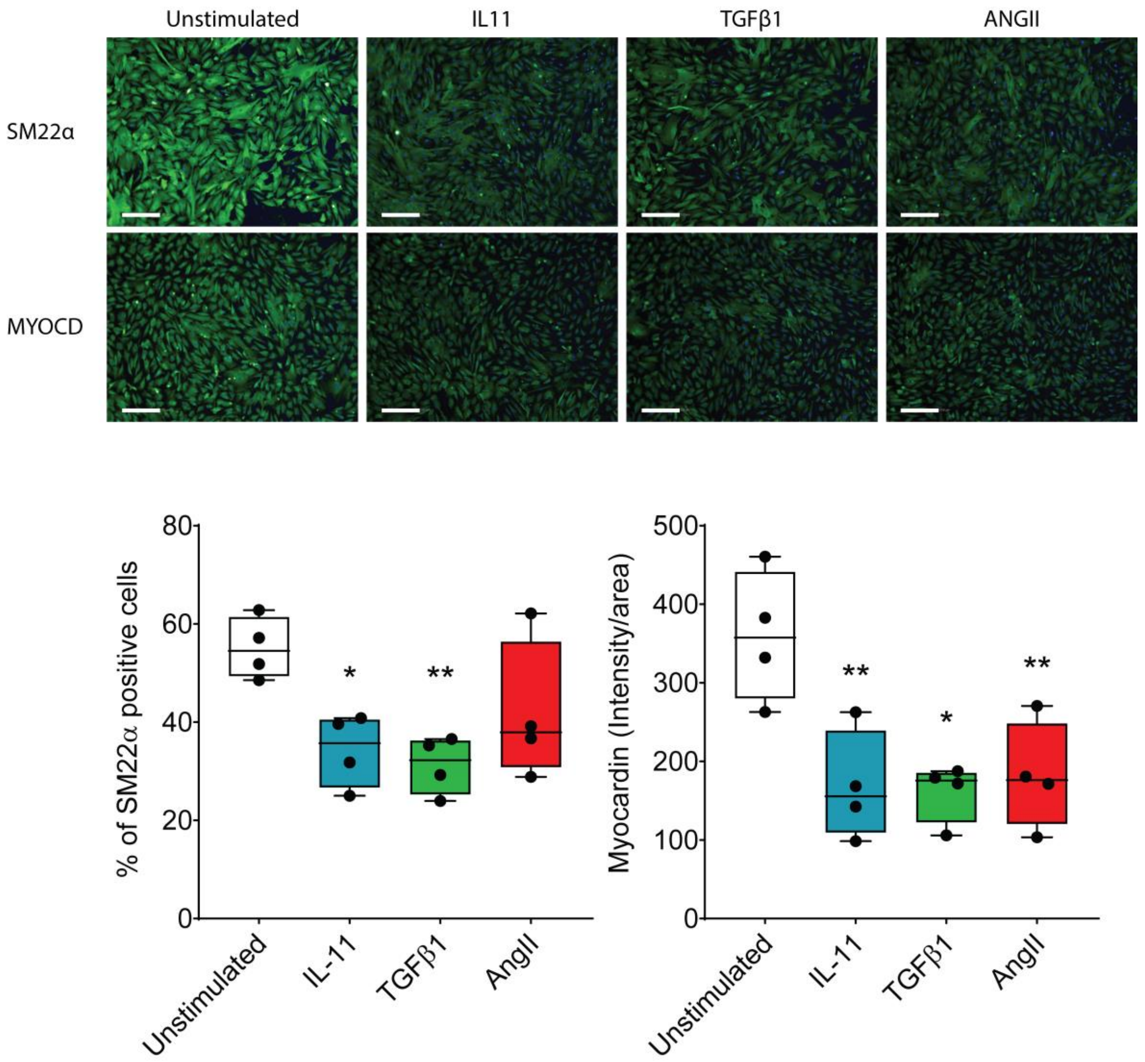

Supplementary Fig. S7. IL11 stimulation in human VSMCs results in reduced expression of transgelin (SM22α) and myocardin (MYOCD) *in vitro*. $SM22^{+ve}$ VSMCs and MYOCD expression by human VSMCs following IL11, TGFβ1 or ANGII stimulation. Representative immunostaining images and Operetta assay results from 4 independent experiments. Statistical analyses by one-way ANOVA with Sidak post hoc tests; data presented as median ± interquartile range (IQR) with whiskers demarcating minimum and maximum values. * $P < 0.05$ and ** $P < 0.01$.

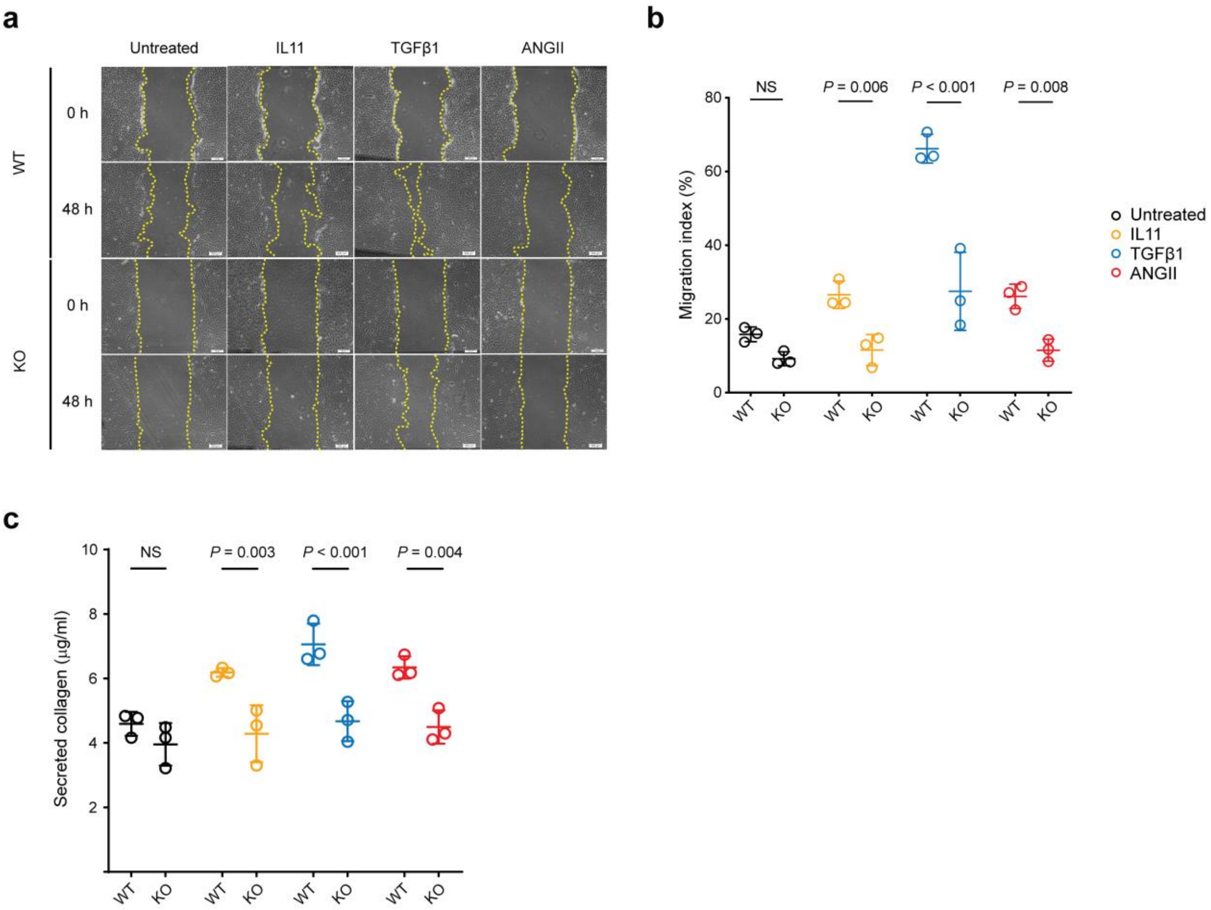

Supplementary Fig. S8. IL11ra1-null VSMCs have attenuated IL11, TGFβ1 or ANGII-induced migration. (**a**) Representative scratch wound assay in a monolayer of *Il11ra1*$^{+/+}$ (WT) and *Il11ra1*$^{-/-}$ (KO) murine aortic VSMCs incubated without stimulus (unstimulated), IL11 (5 ng/ml), TGFβ1 (5 ng/ml) and ANGII (100 nM) for 48h. Scale bar represents 200 µm. (**b**) KO VSMCs were protected against IL11, TGFβ1 and ANGII induced cell migration as assessed by scratch wound assay (*n* = 3 independent experiments). (**c**) Collagen secretion as measured by Sirius red collagen detection assay in culture medium following cytokine stimulation (*n* = 3 independent experiments). Statistical analyses by two-way ANOVA with Sidak multiple comparisons; data presented as mean ± SD.

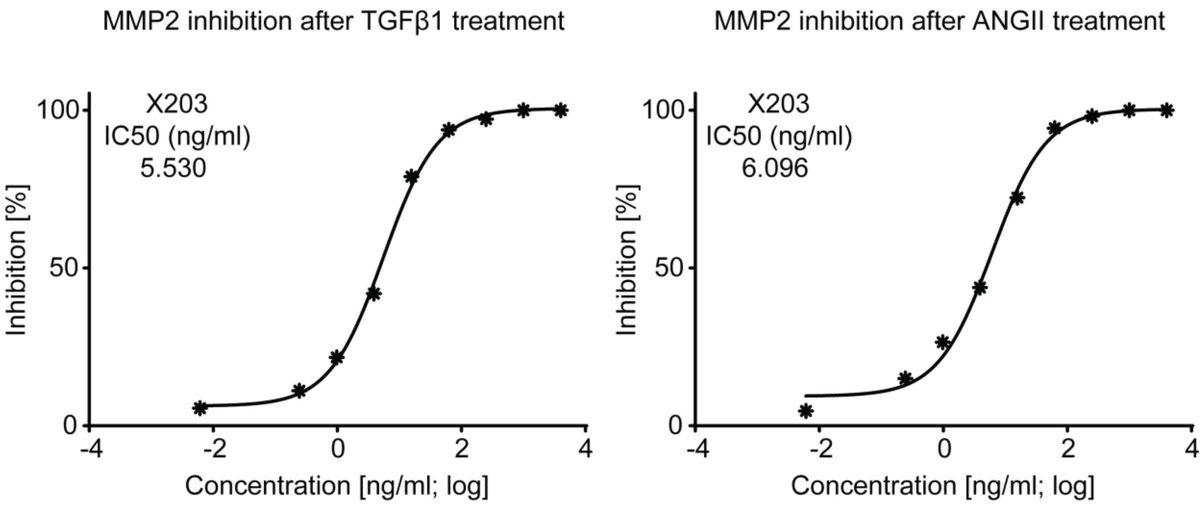

Supplementary Fig. S9. Anti-IL11 (X203) antibodies demonstrate high efficiency in inhibiting MMP2 secretion in VSMCs post TGFβ1 or ANGII stimulation by neutralizing downstream IL11. Primary human VSMC were stimulated with human recombinant TGFβ1 (5 ng/ml) or ANGII (100 nM) in the presence of increasing doses of purified X203 antibodies and culture supernatant was extracted 24h post-stimulation for the quantification of MMP2 concentrations. Inhibition of MMP2 secretion was measured as a percentage of MMP2 concentration compared to TGFβ1 (5 ng/ml) or ANGII (100 nM) stimulation without X203 antibodies.

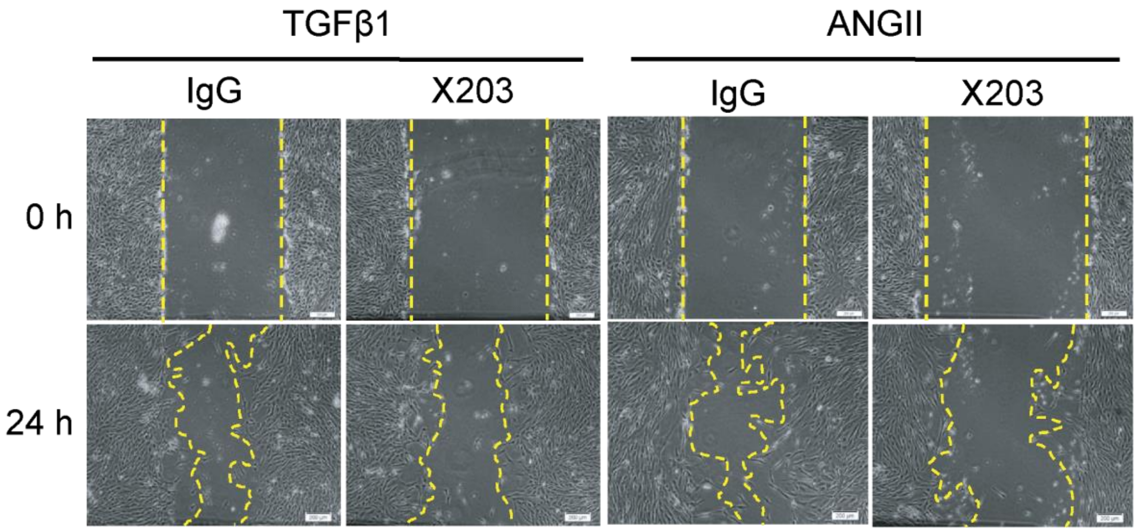

Supplementary Fig. S10. Scratch wound healing assay in human VSMCs treated with anti-IL11 antibodies (X203) inhibits TGFβ1- or ANGII-induced migration. Representative photomicrographs of VSMCs treated with TGFβ1 (5 ng/ml) and ANGII (100 nM) in the presence of either IgG isotype control or X203 antibodies (2 μg/ml) of Fig. 2C. Scale bar represents 200 μm.

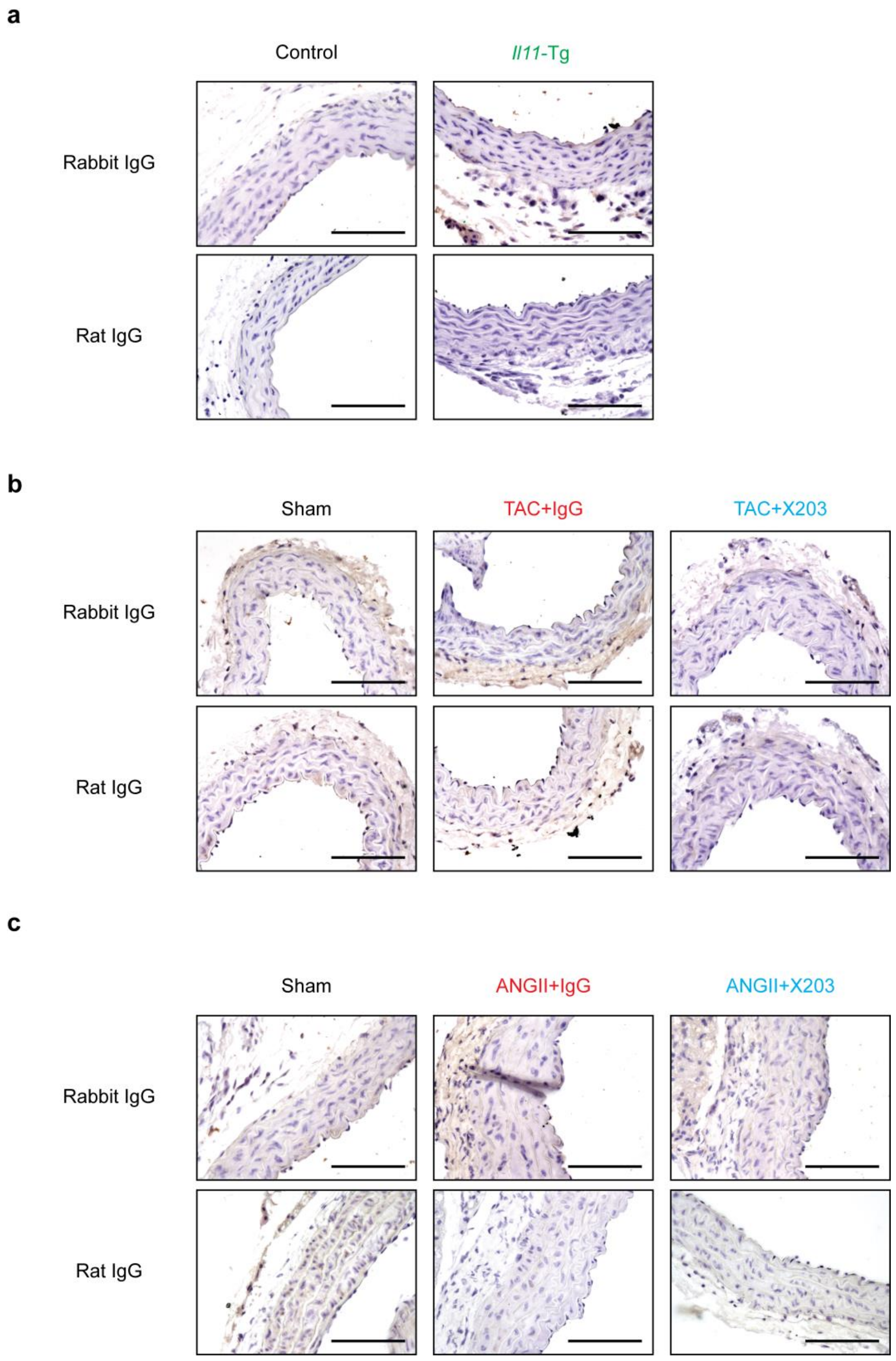

Supplementary Fig. S11. Rabbit and rat isotype controls used for immunohistochemistry in the various models. Respective controls shown in (**a**) Fig 3l, in (**b**) Fig 5l and in (**c**) Fig 7l. Scale bars represent 100µm.

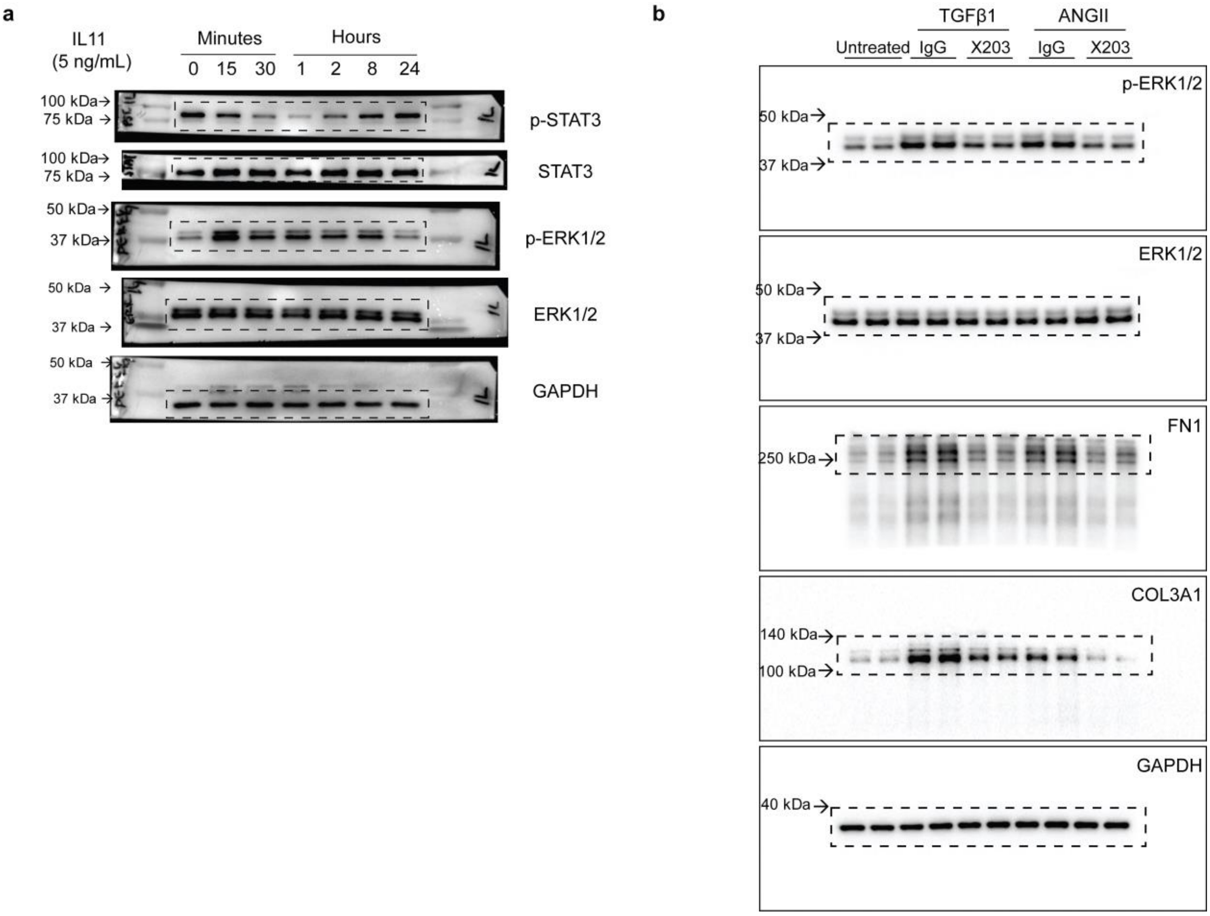

Supplementary Fig. S12. Uncropped Western blots from Figure 2. Immunoblots as shown in (**a**) Fig. 2e and (**b**) Fig. 2f. Cropped inserts shown in main figures are demarcated by dotted box. Samples were derived from the same experiment and blots were processed in parallel.

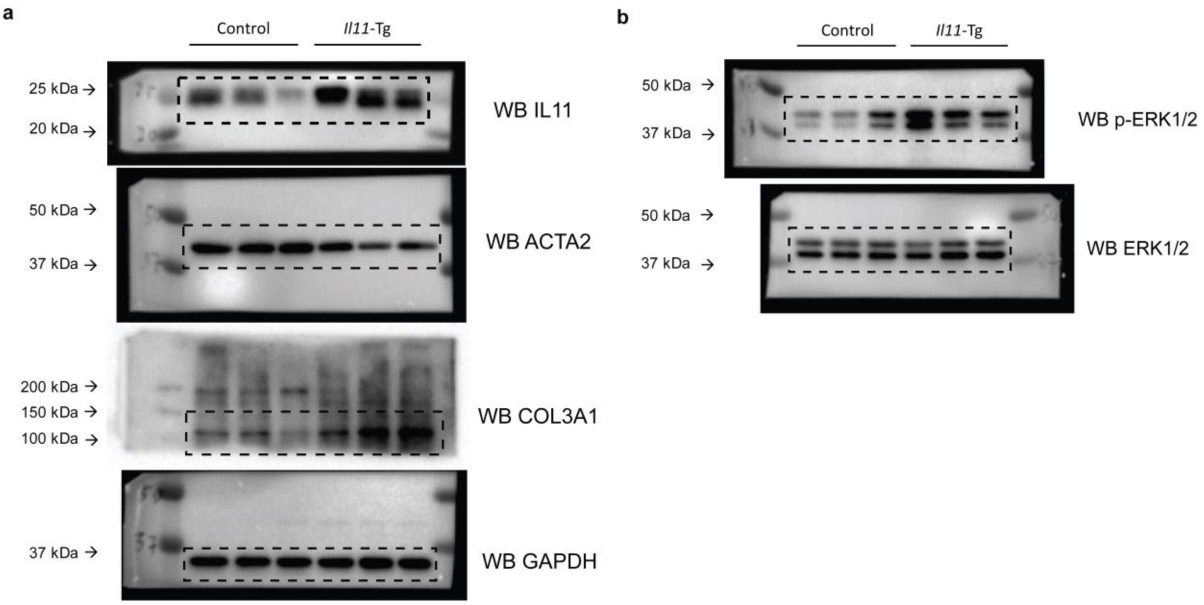

Supplementary Fig. S13. Uncropped Western blots from Figure 4. Immunoblots as shown in (**a**) Fig. 4b and (**b**) Fig. 4c. Cropped inserts shown in main figures are demarcated by dotted box. Samples were derived from the same experiment and blots were processed in parallel.

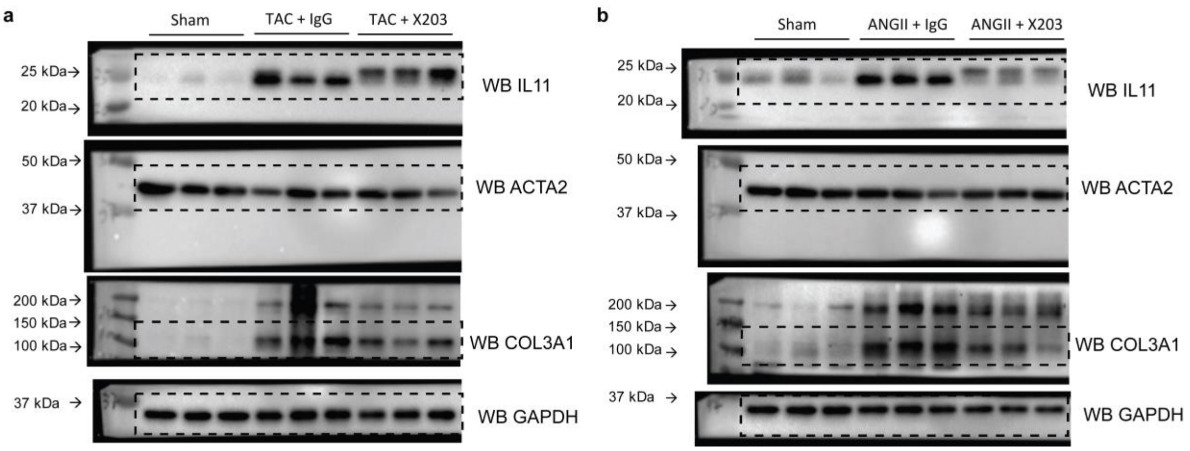

Supplementary Fig. S14. Uncropped Western blots from Figures 6 and 8. Immunoblots as shown in (**a**) Fig 6b and (**b**) Fig. 8b. Cropped inserts shown in main figures are demarcated by dotted box. Samples were derived from the same experiment and blots were processed in parallel.
